# Membrane structure and internalization dynamics of human Flower isoforms hFWE3 and hFWE4 indicate a conserved endocytic role for hFWE4

**DOI:** 10.1016/j.jbc.2023.104945

**Published:** 2023-06-20

**Authors:** Justin C. Rudd, Sibaprasad Maity, James A. Grunkemeyer, Joshua C. Snyder, Sándor Lovas, Laura A. Hansen

**Affiliations:** 1 Department of Biomedical Sciences, Creighton University School of Medicine, Omaha, Nebraska, USA; 2 Department of Surgery, Duke University Medical Center, Durham, North Carolina, USA; 3 Department of Cell Biology, Duke University Medical Center, Durham, North Carolina, USA

**Keywords:** Flower, cell competition, membrane protein structure, molecular dynamics simulations, live-cell imaging, endocytosis

## Abstract

Human Flower (hFWE) isoforms hFWE1-4 are putative transmembrane (TM) proteins that reportedly mediate fitness comparisons during cell competition through extracellular display of their C-terminal tails. Isoform topology, subcellular localization, and duration of plasma membrane presentation are essential to this function. However, disagreement persists regarding the structure of orthologous fly and mouse FWEs, and experimental evidence for hFWE isoform subcellular localization or membrane structure is lacking. Here, we used AlphaFold2 and subsequent molecular dynamics-based structural predictions to construct epitope-tagged hFWE3 and hFWE4, the most abundant human isoforms, for experimental determination of their structure and internalization dynamics. We demonstrate that hFWE3 resides in the membrane of the endoplasmic reticulum (ER), while hFWE4 partially colocalizes with Rab4-, Rab5-, and Rab11-positive vesicles as well as with the plasma membrane. An array of imaging techniques revealed that hFWE4 positions both N- and C-terminal tails and a loop between second and third TM segments within the cytosol, while small (4–12aa) loops between the first and second and the third and fourth TM segments are either exposed to the extracellular space or within the lumen of cytoplasmic vesicles. Similarly, we found hFWE3 positions both N- and C-terminal tails in the cytosol, while a short loop between TM domains extends into the ER lumen. Finally, we demonstrate that hFWE4 exists only transiently at the cell surface and is rapidly internalized in an AP-2- and dynamin-1-dependent manner. Collectively, these data are consistent with a conserved role for hFWE4 in endocytic processes.

Flower (FWE) proteins are small, highly conserved, putative membrane proteins produced from a single alternatively spliced *FWE* (also known as *CACFD1*) gene. The human *hFWE* locus encodes four isoforms: hFWE1, hFWE2, hFWE3, and hFWE4. It has been proposed that in human cells, as in *Drosophila*, differential expression of hFWE isoforms in neighboring cells is necessary and sufficient to elicit cell competition, a process whereby less-fit cells are non-autonomously eliminated from heterogenous tissues (1, 2, 3). In competition between human cancer cells, isoforms exhibit functional redundancy, with epithelial cells or fibroblasts expressing high levels of hFWE1 or hFWE3 undergoing cell death only when neighbored by epithelial cells expressing high levels of hFWE2 or hFWE4 (1). While the molecular mechanisms mediating this hFWE-dependent competition in human cells are entirely unknown, seminal work in *Drosophila* proposed that positioning of unique C-terminal tails in the extracellular space may facilitate the “fitness-sensing” that drives this process (2, 3). In theory, this mechanism relies on integration of FWE isoforms into the plasma membrane as well as membrane topologies that position C-terminal tails in the extracellular space. Intriguingly, work implicating FWE isoforms in endocytic processes suggested that the canonical FWE isoform in both fly (4, 5, 6) and mouse (7) assumes a four TM structure that positions both N- and C-terminal tails in the cytosol. This structure imparts direct calcium channel activity during synaptic vesicle endocytosis at neuromuscular junctions (4, 5, 6) and facilitates calcium-regulated processes such as cytotoxic granule endocytosis in cytotoxic T-lymphocytes (7) independent of direct calcium channeling. While both FWE-mediated cell competition and FWE-dependent endocytic processes rely on the integration of FWE into the plasma membrane, these prior studies present conflicting membrane topologies with significant implications for the molecular function of FWE isoforms. Any mechanistic investigation into FWE-dependent processes in human cells will be greatly aided by work that defines subcellular localization and membrane topology of human hFWE isoforms.

Before undertaking such an analysis, we sought to identify expression levels of *hFWE* isoforms across different tissue types. Exon and isoform level expression data from both Illumina sequencing and recently deposited long-read Oxford nanopore-technology-based sequencing (8) shows that hFWE3 and hFWE4 are the most abundant isoforms in all human tissues surveyed in the GTEx Biobank (Fig. S1, *A*–*D*). These isoforms share exons 1, 2, and 4, exhibit variable inclusion of exon 3 and utilize the second of two open reading frames in C-terminal exon 6 (Fig. 1*A*). Hydropathy-based topological prediction (1) suggested that this produces a 2 TM and a 3 TM structure for hFWE3 and hFWE4, respectively. These topological models position the N and C-terminal tails of hFWE4 on opposing sides of the membrane, as has been proposed for *Drosophila* isoforms (2, 3). Additionally, these models suggest that amino acids encoded by shared exon four are transmembrane in hFWE3 but part of the extracellularly exposed C-terminal tail of hFWE4. These topologies allow each isoform to display a unique C-terminal tail which has been proposed as the basis for cell-cell fitness sensing during FWE-mediated cell competition. However, experimental evidence from mouse cytotoxic T-lymphocytes (CTLs) suggests that the canonical mFWE2 isoform, which shares 92.4% amino acid sequence identity with hFWE4, assumes a four TM structure that positions the N and C tails within the cytosol. As these terminal domains harbor AP-2 adaptor complex binding motifs, their cytosolic exposure is essential for proper clathrin-mediated endocytosis (CME) of the molecule from the plasma membrane (7). No direct experimental evidence exists for a particular membrane topology of any of the four human isoforms, despite its importance for understanding the molecular function of hFWE. Additionally, as hFWE3 is less structurally conserved across species than the canonical hFWE4, it is difficult to draw conclusions about its membrane structure. Experimental evidence clarifying cytosolic or extracellular exposure of key hFWE epitopes is needed to properly validate the structure of these proteins.

**Figure 1:**
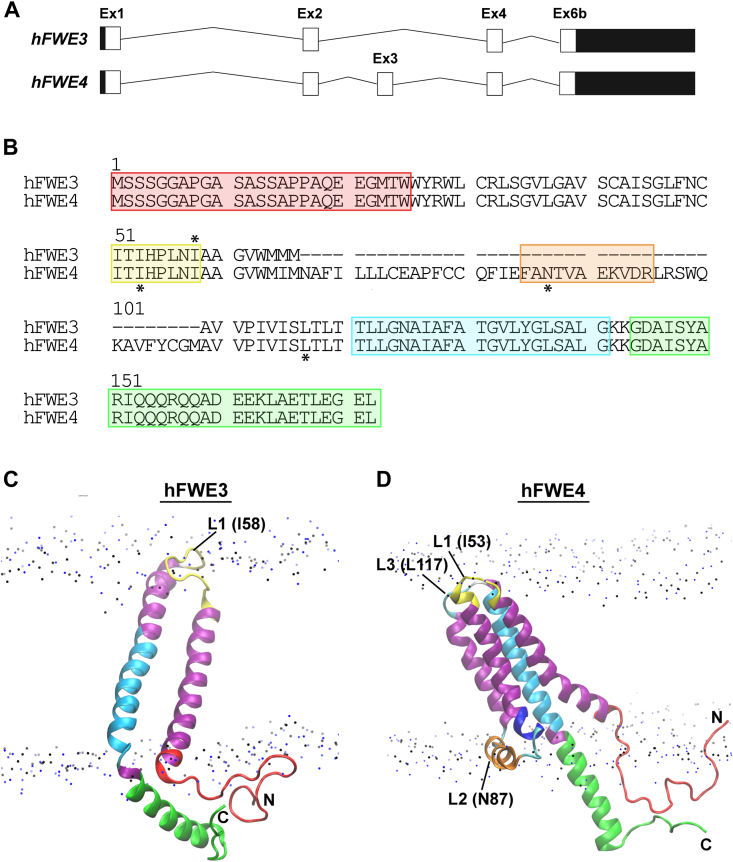
**Primary and tertiary structures of hFWE3 and hFWE4.***A*, exon structure of *hFWE3* and *hFWE4* transcripts. 5′ and 3′ UTR are indicated by *black boxes*. *B*, sequence alignment of hFWE3 and hFWE4. The two proteins are identical at amino acid residues 1 to 66 and 109 to 172 (numbering refers to hFWE4). hFWE3 is a splice variant of hFWE4 missing amino acid residues of 67 to 108 (exon 3). *Colored boxes* indicate the location of synthetic peptide fragments generated in Table 1. *Red*, N-terminal tail; *yellow*, loop 1 (L1); *cyan*, transmembrane two region, *green*, C-terminal tail. Starred residues indicate those immediately preceding epitope tag insertions in loop-tagged hFWE3 and hFWE4 constructs. *C* and *D*, representation of the lowest energy structure of hFWE3 (*C*) and hFWE4 (*D*) on PC1-PC2 free energy landscape (see Fig. S4) embedded in a POPC phospholipid bilayer. For the POPC phospholipid membranes only the N and P atoms are presented as *dots*. Annotations denote the location of amino acid residues immediately preceding epitope tags in chimeric constructs generated for experimental validation (hFWE3 - N, L1 (I58), and *C*; hFWE4 – N, L1 (I53), L2 (N87), L3 (L117), and *C*). *Color* coding of structures in (*C* and *D*) corresponds to the color coding of residues in (*B*).

Plasma membrane integration, and the duration with which FWE molecules exist at the plasma membrane, is likely of critical importance for both their role as fitness sensors during cell competition as well as their endocytic function. In mouse, plasma membrane presentation has been experimentally validated for the canonical mFWE2, which displays highly polarized and transient surface exposure at the immunological synapse in CTLs (7). Evidence for plasma membrane integration of *Drosophila* FWE isoforms is well documented, with immunofluorescence data supporting this localization to varying degrees in both neurons (4, 5, 6, 9) and epithelia (2, 3, 10). However, AlphaFold2 (AF2) (11, 12) modeling suggests that *Drosophila* isoforms are highly structurally similar to one another, with unique C-terminal tails not impacting the predicted folding of the conserved four-helix core domain. Interestingly, hFWE4 retains this core domain while hFWE3 does not. While plasma membrane integration for all hFWE isoforms has been hypothesized (1), this requires experimental validation considering the predicted structural differences across these proteins.

Here, we use AlphaFold2 (11, 12) in concert with molecular dynamics simulations to generate structural models for experimental validation of hFWE3 and hFWE4 membrane structure. Utilizing an array of subcellular imaging techniques, our experiments revealed that hFWE4, but not hFWE3, is trafficked to the plasma membrane, where it assumes a four TM structure with two small extracellular loops and cytosolic N- and C-terminal tails. hFWE3 is resident in the membrane of the endoplasmic reticulum but similarly positions N- and C-terminal tails into the cytosol. Functional imaging and biochemical assays demonstrated that hFWE4, like its murine ortholog, is rapidly internalized from the plasma membrane through a mechanism reliant on functional AP-2 binding motifs conserved at the N- and C-terminal region of the protein. Our structural data suggest that the display of hFWE-isoform specific C-terminal tails across neighboring epithelia is unlikely to serve as a mechanism for initiating hFWE-mediated competition and additionally illustrate a conserved mechanism of hFWE4 internalization that could regulate the presentation of yet undefined mediators of these competitive interactions.

## Results

### Structural prediction of hFWE3 and hFWE4

To reconcile discrepancies between hydropathy-based topological predictions of hFWE structure (1) and the experimentally supported structure of the murine mFWE2 ortholog (7), AF2 and subsequent MD simulations were employed to produce robust structural models for hFWE3 and hFWE4. Amino acid sequences for hFWE3 and hFWE4 (Fig. 1*B*) were submitted to AF2, which predicted multi-helical structures for both proteins (Fig. S2). hFWE3 is composed of an N-terminal random meander tail, a two-helix bundle that is connected by a short helical loop (L1), and a C-terminal helical tail (Figs. 1*C* and S2*A*, top). The predicted structure of hFWE4 includes an N-terminal random coil tail, a four-helix bundle, and a C-terminal helical tail. In hFWE4, helices 1 and 2, and helices three and four are connected by β-bends (referred to as loop 1 (L1) and loop 3 (L3), respectively), whereas helices two and three are connected by a short helical loop (referred to as loop 2 (L2)) (Figs. 1D and S2*B*, top). The identical sequence regions of the two proteins are predicted to have the same secondary structures, except for the first loop that is helical in hFWE3 and β-bend in hFWE4.

### Refining hFWE3 and hFWE4 structural predictions using MD simulations

To validate and refine these predicted structures, two-step MD simulations were performed (see Supporting Information and Figs. S3–S7). MD simulations confirmed that hFWE3 assumed a two-pass TM conformation, while hFWE4 assumed a four-pass TM conformation as shown in renderings of lowest energy structures (Figs. 1, *C* and *D* and S5 and S6). For hFWE3, this positioned N- and C-terminal tails on one side of the membrane, and loop 1 (L1) connects the two-helix bundles on the other. For hFWE4, N- and C-terminal tails and loop 2 (L2) occupied the same side of the membrane, while L1 and L3 were on the opposite side.

### CD spectropolarimetry analysis of protein fragments

We next sought to validate the secondary structure of critical regions of hFWE3 and hFWE4 using CD spectropolarimetry. Synthetic fragments corresponding to N- (NT) and C-terminal tails (CT), surface loops L1 and L2, and transmembrane helix 4 (TM4) (see color coding in Fig. 1, *B*–*D*) were synthesized for CD analysis. Detailed CD spectropolarimetry data are compiled in Table 1 and Fig. S7. In agreement with simulation data, the secondary structure of the NT peptide was a mixture of helix, turns, sheet and unordered conformation, while the CT peptide had a more pronounced helical conformation with a mixture of sheet and unordered conformations. CD spectra of L1 peptide indicated stabile β-turn/bend conformations, while L2, which is laying on the membrane surface, had a strong tendency to form helical conformation. Collectively, these data supported a two-pass TM structure for hFWE3 and a four-pass TM structure for hFWE4, both of which position N- and C-tails on the same side of the membrane, consistent with the AF2 predictions. These robust structural models subsequently informed our strategy for the experimental determination of hFWE3 and hFWE4 structures in human cells.

**Table 1:** Fraction of secondary structure of peptides

Peptide	% TFE	Helix^a^	β- sheet	Turns	Unordered	Total
NT	0	0.11	0.29	0.23	0.36	0.99
L1	0	0.11	0.26	0.25	0.37	0.99
	15	0.11	0.29	0.27	0.34	1.0
	30	0.10	0.27	0.26	0.36	0.99
	45	0.06	0.35	0.22	0.36	0.99
L2	0	0.07	0.27	0.21	0.44	0.99
	15	0.10	0.27	0.23	0.39	0.99
	30	0.29	0.22	0.19	0.30	1
	45	0.65	0.15	0.04	0.16	0.99
TM4^b^	0	0.29	0.20	0.2	0.29	0.98
	30	0.59	0.08	0.1	0.23	1.0
	45	0.47	0.12	0.13	0.25	0.99
CT	0	0.16	0.19	0.09	0.36	1

Secondary structure of peptides were determined by CD spectropolarimetry at increasing concentrations of TFE. Composition of secondary structures were obtained by deconvoluting CD spectra with CDSSTR algorithm using Dichroweb server using Basis set 4 as reference.

a Helix is a mixture α- and 3_10_-helices.

b Initially, it was dissolved in 33% ACN in water.

### Characterizing subcellular localization of hFWE3 and hFWE4

**Figure 2:**
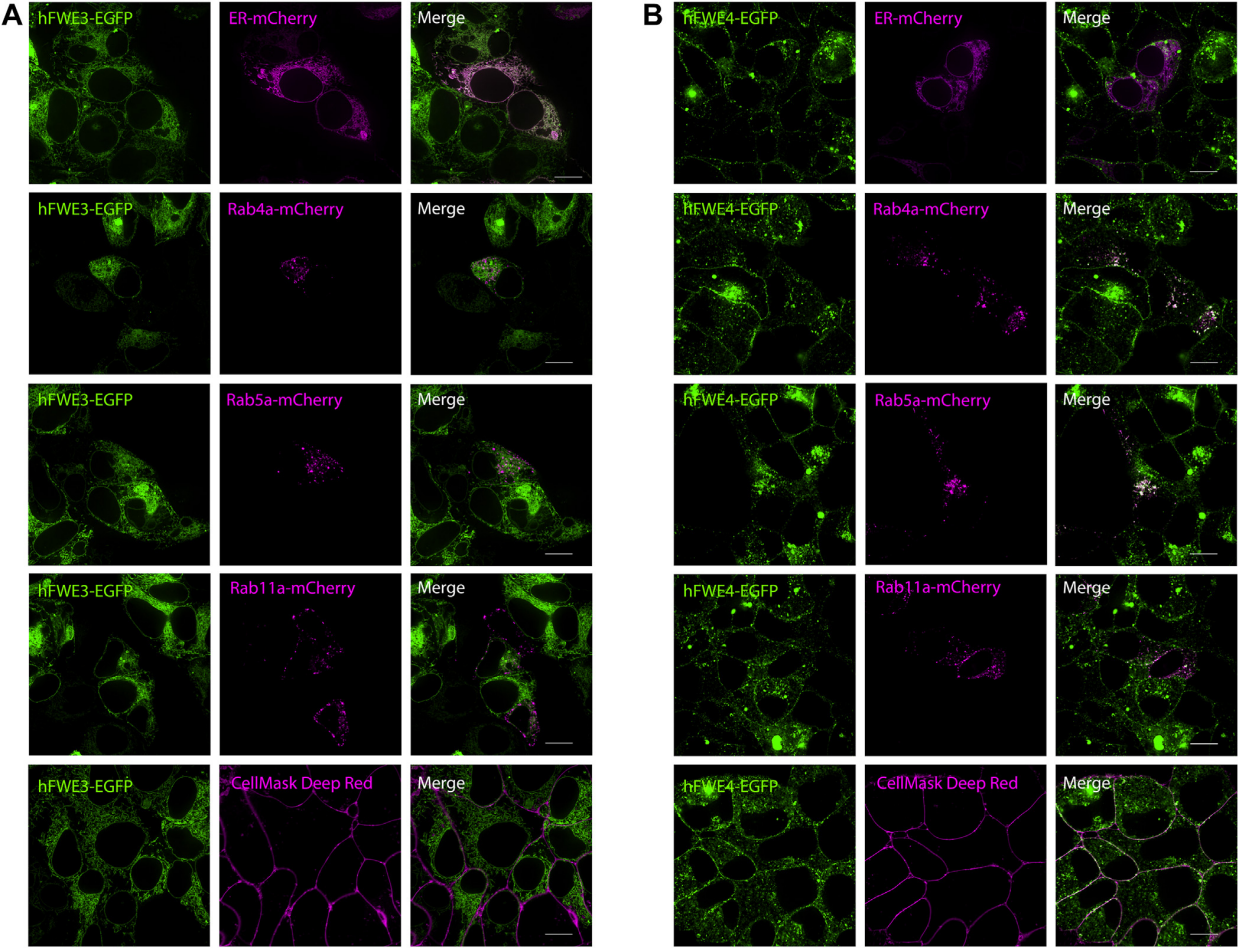
**hFWE3 and hFWE4 reside in different subcellular compartments.** HEK293 cells stably expressing hFWE3-EGFP (*A*) or hFWE4-EGFP (*B*) were transfected with indicated mCherry fusion constructs or subjected to plasma membrane labeling with CellMask *Deep Red* and live imaged for native EGFP (*green*) and mCherry or far-red fluorescence (*magenta*). Shown are single Z-slices from representative 100× live cell confocal images, scale bar = 10 μm.

To investigate the subcellular distribution and membrane integration of hFWE isoforms in human epithelial cells, stable hFWE3-EGFP or hFWE4-EGFP expressing HEK293 cells were imaged, revealing markedly different patterns of localization for hFWE3 and hFWE4 (Fig. 2, *A* and *B*, left-hand columns). hFWE3 displayed perinuclear and contiguous reticular signal characteristic of endoplasmic reticulum (ER) resident proteins (Fig. 2*A*), while hFWE4 produced both punctate cytoplasmic and plasma membrane signal (Fig. 2*B*). To further characterize subcellular hFWE localization, mCherry chimeras targeting various intracellular structures were transiently expressed in stable hFWE3-EGFP and hFWE4-EGFP HEK293 cells. hFWE3-EGFP signal exhibited significant colocalization with the endoplasmic reticulum, while hFWE4-EGFP colocalized partially with markers of early endosomes (Rab4a, Rab5a) and recycling endosomes (Rab11a) (Fig. 2, *A* and *B*). hFWE4-EGFP but not hFWE3-EGFP also produced steady-state plasma membrane signal as evidenced by colocalization with CellMask dye, suggesting that some hFWE4 was present at the plasma membrane (Fig. 2*B*) as previously reported for both fly and mouse orthologs. Neither isoform showed significant colocalization with lysosomal targeting Rab7a, lysosomes, or Golgi (Fig. S8). These data suggest that hFWE3 and hFWE4 isoforms are targeted to different subcellular compartments in human epithelial cells with hFWE3 remaining embedded in the ER membrane, while hFWE4 is trafficked to the plasma membrane.

### Experimental determination of hFWE isoform structure at the cell surface

**Figure 3:**
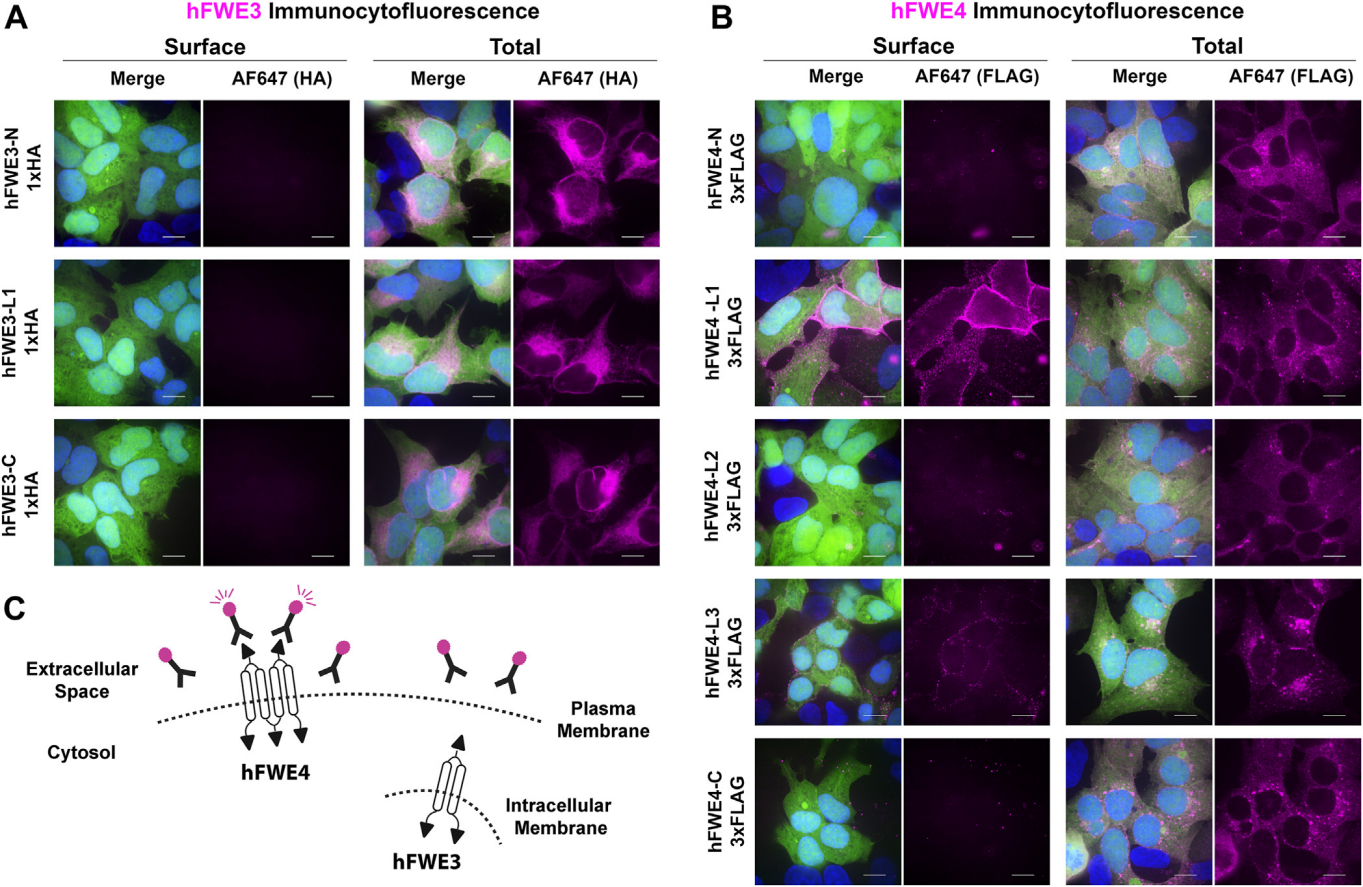
**Plasma membrane integrated hFWE4 assumes a four transmembrane pass structure with cytosolic N and C termini.** Labeling of surface-exposed epitopes (*A*; *left panels*, *B*; *left panels*) in HEK293 cells stably coexpressing non-fused EGFP and (*A*) hFWE3 or (*B*) hFWE4 harboring epitope tags at indicated positions was performed by pulsing cells with the primary antibody on ice for 45 min prior to fixation and secondary antibody labeling. Labeling of total ectopic hFWE (*A*; *right panels*, *B*; *right panels*) was performed by fixing and permeabilizing cells prior to primary incubation. Shown are representative 100× confocal maximum intensity projections of immunolabeled hFWE (*magenta*), native EGFP (*green*), and Hoechst (*blue*), scale bar = 10 μm. *C*, schematic of hypothesized hFWE4 plasma membrane structure which presents N-terminal tail, L2, and C-terminal tail cytosolically, while L1 and L3 are exposed to the cell surface. hFWE3 is absent from the plasma membrane. Schematic shows hFWE3 orientation on intracellular membranes.

Informed by our modeling data, a series of epitope-tagged hFWE3 (HA-tagged N, L1, or C) and hFWE4 (3xFLAG-tagged N, L1, L2, L3, or C) lentiviral expression constructs were generated to allow for experimental validation of the proposed membrane structure of both hFWE isoforms. Because the plasma membrane localization of hFWE isoforms was expected to be the most functionally important site for their competitive and endocytic roles, we first sought to experimentally determine any cell surface–exposed regions. Live cell immunofluorescence of epitope tagged-hFWE expressing HEK293 cells revealed no detectable surface labeling of hFWE3 regardless of the position of the 1xHA tag (Fig. 3*A*). When fixed and permeabilized prior to antibody labeling, hFWE3 expressing cells instead displayed the characteristic ER-associated subcellular localization observed in live imaging of hFWE3-EGFP fusion proteins (Fig. 3*A*, total). Intriguingly, hFWE4 surface labeling did not yield a detectable signal when the 3xFLAG epitope was positioned at the N or C-terminus or when situated in the middle of L2, despite these expression constructs yielding expected subcellular distribution under fixed and permeabilized conditions. However, when the 3xFLAG was positioned at the L1 or L3, clear, punctate surface labeling was evident (Fig. 3*B*). Importantly, surface labeling occurred in the absence of propidium iodide incorporation, demonstrating a lack of membrane permeability (Fig. S9). Together, these data suggested that hFWE4, but not hFWE3, is present at the plasma membrane and that hFWE4 likely assumes a four TM structure with N-terminal tail, L2 and C-terminal tail positioned cytosolically, while L1 and L3 are exposed to the extracellular environment (Fig. 3*C*).

### Experimental determination of hFWE isoform structure on intracellular membranes

While surface labeling demonstrated that L1 and L3 for hFWE4 are exposed to the extracellular space, the topology of hFWE3 in the ER membrane remained unclear, and experimental evidence for the cytosolic localization of hFWE4 N- and C- termini as well as L2 was lacking. To solidify these topologies, live imaging of “Frankenbodies”, genetically encoded, fluorescent protein-coupled, single chain variable fragments that target HA or FLAG epitopes (13, 14), was performed. Upon their expression in a cell coexpressing an HA- or FLAG-tagged protein, Frankenbodies rapidly assume the subcellular distribution of their target. While Frankenbodies diffuse freely through the cytoplasm and can be trafficked into more permeable organelles such as the nucleus to bind their cognate epitope, we reasoned that they would be prevented from accessing epitopes protected within the lumen of the ER or endosomes.

**Figure 4:**
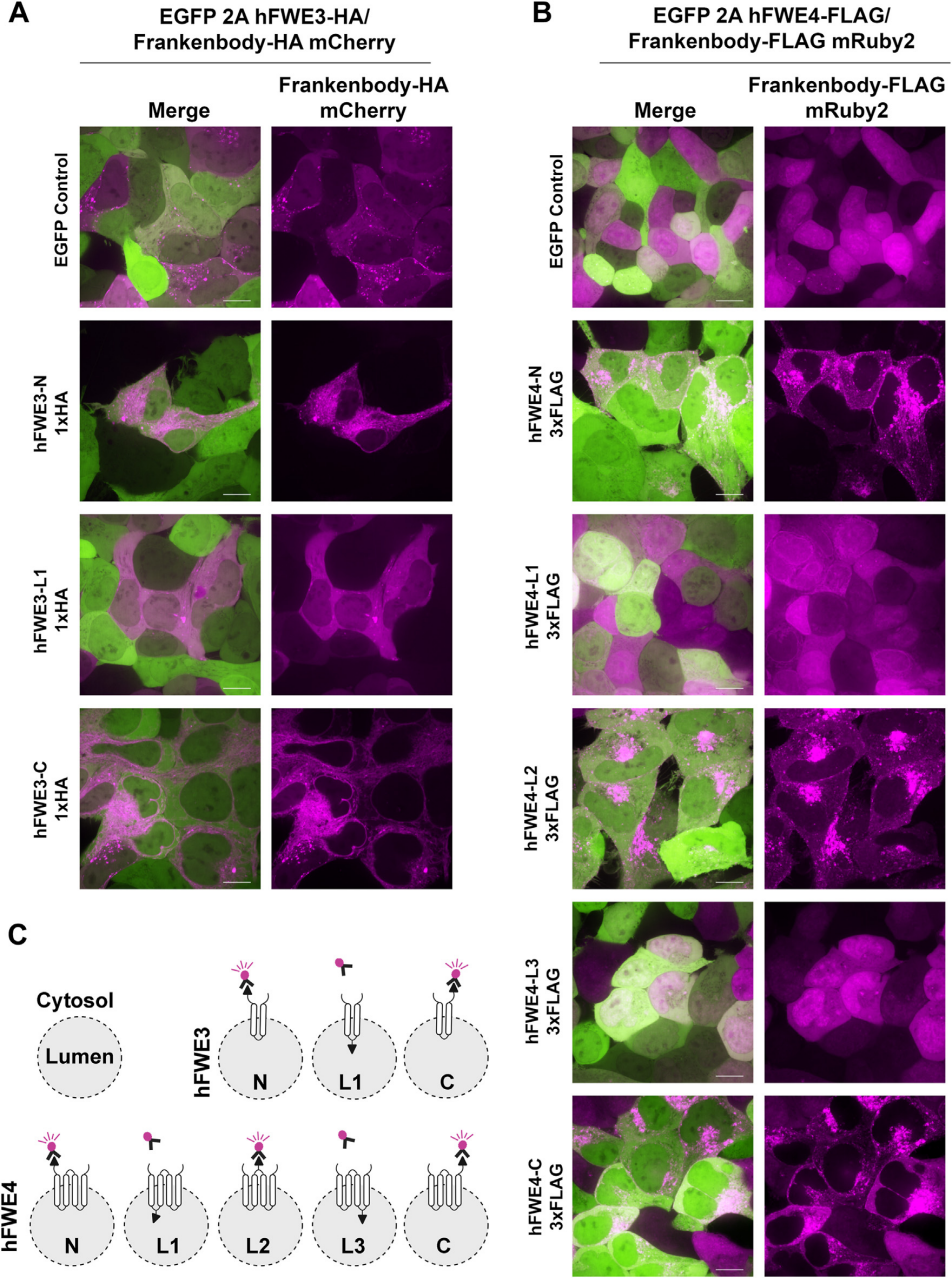
**Frankenbodies validate hFWE isoform structure on organellar and vesicular membranes.** HEK293 cells stably expressing EGFP alone, or coexpressing non-fused EGFP and hFWE3 (*A*) or hFWE4 (*B*) harboring epitope tags at indicated positions were transduced with lentivirus encoding HA- or FLAG-targeting Frankenbodies. Transduced cells were live imaged for native EGFP (*green*) and mCherry/mRuby2 fluorescence (*magenta*). Representative max intensity projections of central Z-slices are presented from 100× live cell confocal images, scale bar = 10 μm. *C*, schematic overview of hFWE isoform structure on intracellular membranes, with hFWE4 assuming a four transmembrane pass structure and hFWE3 assuming a two transmembrane pass structure. Both isoforms position N and C termini cytosolically.

To test whether we could discriminate between motifs exposed to the cytosol or protected within vesicle lumens, subcellular live imaging of HEK293 cells expressing EGFP alone without an HA or FLAG-tagged hFWE isoform revealed mCherry and mRuby2 signal from the HA and FLAG Frankenbodies, respectively, diffusely labeled the entire cell, with only slight aggregation observed for mCherry (Figs. 4, *A* and *B* and S10, *A* and *B*, top panels). In hFWE3 expressing cells, the mCherry signal derived from the HA-Frankenbody assumed the characteristic ER pattern when hFWE3 harbored N- or C-terminal HA tags (Figs. 4*A* and S10*A*). However, the distribution of HA-Frankenbody associated mCherry signal remained diffuse when in cells expressing hFWE3 bearing an HA tag at L1, indicating epitope inaccessibility (Figs. 4, *A* and *C* and S10, *A* and *C*). Similarly, in cells expressing hFWE4 harboring 3xFLAG at the N-terminus, C-terminus, or in L2, mRuby2 signal associated with the FLAG-Frankenbody assumed the expected vesicular hFWE4 subcellular localization with clear plasma membrane association (Figs. 4*B* and S10*B*). Predictably, FLAG-Frankenbody associated mRuby2 signal diffusely labeled the entire cell when hFWE4 was 3xFLAG tagged at either L1 or L3, which are anticipated to be either extracellular or protected within endosomal lumens (Figs. 4, *B* and *C* and S10, *B* and *C*).

**Figure 5:**
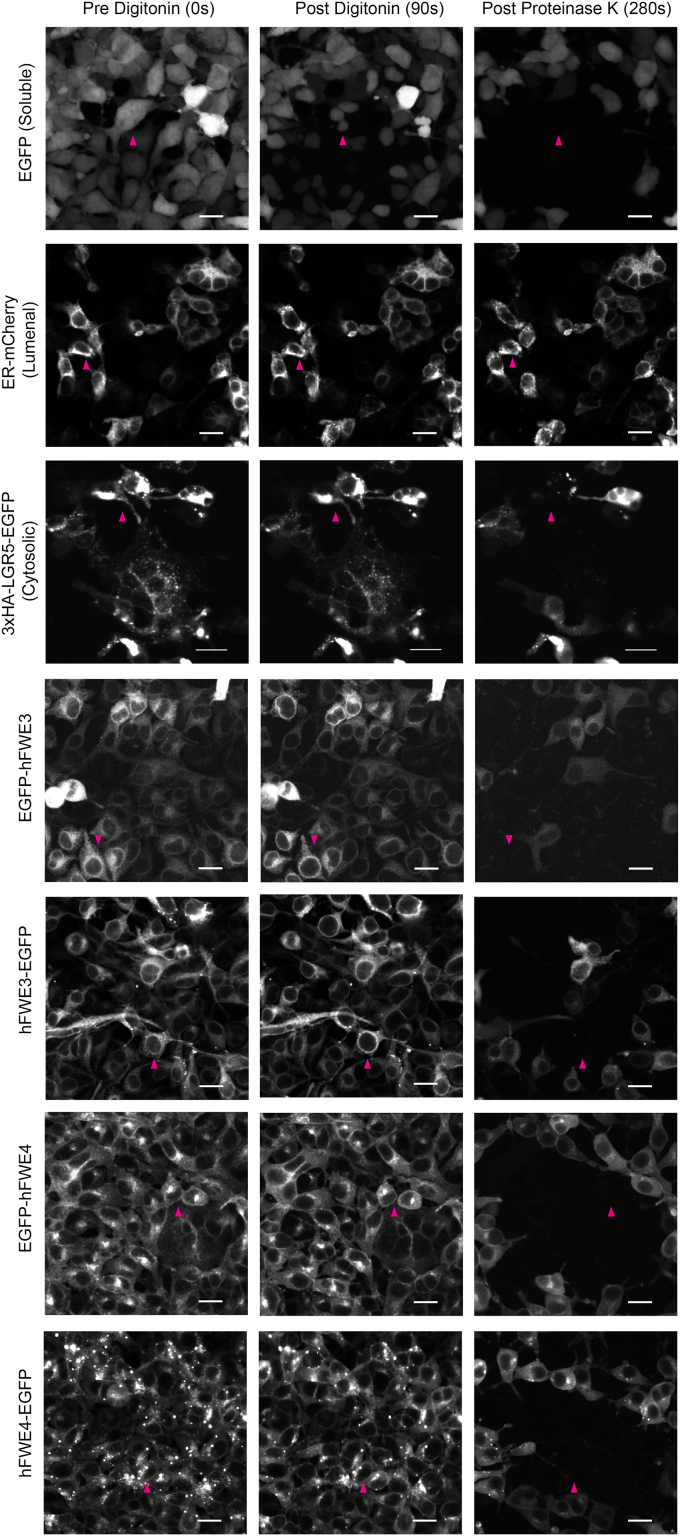
**Fluorescence protease protection confirms cytosolic positioning of N and C terminal tails of hFWE3 and hFWE4.** HEK293 stably expressing EGFP, EGFP-hFWE3, hFWE3-EGFP, EGFP-hFWE4, hFWE4-EGFP, or transiently transfected with 3xHA-LGR5-EGFP or mCherry-ER-3 were incubated in the presence or absence of 80 μg/ml digitonin for 90 s before removal and incubation in KHM buffer with or without 50 μg/ml proteinase K. 20× live confocal imaging at 1 s intervals for native EGFP or mCherry was initiated upon addition of digitonin and continued out to 400 s. Confocal images presented are representative of t = 0, 90, and 280 s time points for each experimental group, scale bar = 20 μM (N ≥ 3 independent timelapses per construct). *Red arrowheads* indicate representative cells across the three timepoints.

The cytosolic positioning of N- and C- terminal tails for both hFWE3 and hFWE4 was further supported in FPP assays utilizing hFWE3 and hFWE4 bearing either N- or C-terminal EGFP fusions. FPP relies on selective permeabilization of the plasma membrane with digitonin which leaves intracellular membranes unperturbed. Subsequent proteinase K treatment is applied to cells that digests cytosolically exposed fluorescent proteins leading to rapid signal decay, while fluorescent markers protected in the lumen of intracellular organelles resist degradation (15). Upon digitonin treatment of HEK293 cells, cytosolic EGFP diffused rapidly from a subset of cells and was further degraded by proteinase K treatment. Additionally, EGFP fused to the cytosolic C-terminus of LGR5 rapidly decayed upon proteinase K treatment, and while partial permeabilization of ER membranes can occur in digitonized cells, mCherry protected within the ER lumen persisted after proteinase K addition. These controls indicated selective permeabilization of only the plasma membrane (Fig. 5). In cells expressing hFWE-EGFP fusion constructs, proteinase K treatment led to rapid degradation of EGFP signal regardless of N- or C-terminal position on either hFWE3 or hFWE4 (Fig. 5, *A* and *B*). These data support the conclusion that at both plasma membrane and intracellular membranes, hFWE4 exists in a four TM conformation that positions both N- and C- terminal tails cytosolically. While hFWE3 is not trafficked from the ER membrane, both Frankenbody and FPP assays indicate that it assumes a two transmembrane domain structure that positions N- and C- terminal tails cytosolically and likely exposes a small loop into the lumen of the ER.

### Clathrin-mediated endocytosis facilitates rapid internalization of hFWE4 from the cell surface

**Figure 6:**
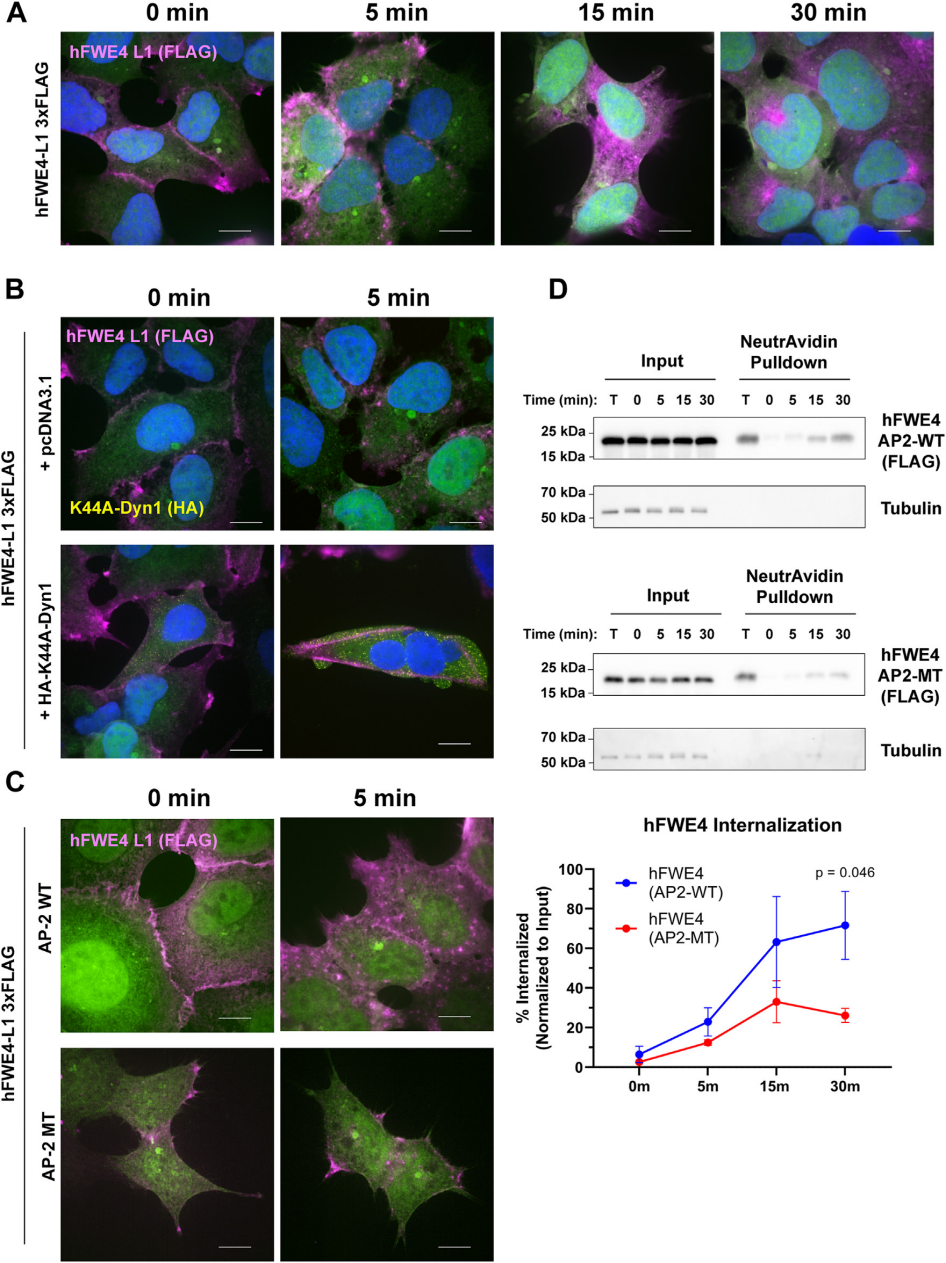
**Rapid internalization of hFWE4 from the plasma membrane is dependent on functional AP-2 motifs and dynamin1-mediated vesicle scission.***A*, HEK293 stably coexpressing non-fused EGFP (*green*) and hFWE4-L1 3xFLAG were pulsed with FLAG antibody for 45 min on ice, washed, chased for 0, 5, 15 or 30 min at 37 °C, and then fixed for fluorescent tyramide based detection (*magenta*) and Hoechst nuclear labeling (*blue*). *B*, HEK293 stably coexpressing non-fused EGFP and hFWE4-L1 3xFLAG were transfected with either pcDNA3.1 or Dyn1-K44A-HA prior to antibody pulse and 0 or 5 min chase as described above. Following tyramide development, Dyn1-K44A-HA was labeled with anti-HA (*yellow*). *C*, HEK293 stably coexpressing non-fused EGFP and either wild-type hFWE4-L1 3xFLAG, or AP-2 motif mutant hFWE4-L1 3xFLAG were pulsed with FLAG antibody and chased for 0 or 5 min before tyramide based detection as described above. *A*−*C*, single Z-slices from representative 100× confocal imaging for native EGFP (*green*), immunolabeled hFWE4 (*magenta*) at indicated time points from each, scale bar = 10 μm. *D*, representative immunoblots from cell surface biotinylation and internalization assays performed in AP2-WT (*top panels*) and AP-2MT (*bottom panels*) hFWE4 expressing HEK293 cells. T = unstripped samples representing total surface biotinylated protein; 0 min = samples subject to Mesna stripping immediately following biotinylation; 5 to 30 min = samples chased at 37 °C for indicated time points to allow internalization, then Mesna stripped to remove remaining biotin from protein remaining at the cell surface. Tubulin was used as a cytosolic control. Input-normalized FLAG signal at each chase timepoint was compared to total surface (T) signal as a readout of the fraction of internalized hFWE4. Graph presents densitometric data from N = 3 replicates, statistical significance was determined using a two-way ANOVA with post-hoc Holm-Sidak correction).

With extracellular labeling of L1 tagged hFWE4 providing a robust method to specifically detect surface exposed hFWE4, we sought to determine the duration with which hFWE4 exists at the plasma membrane in human cells using pulse-chase live cell immunofluorescence. In HEK293 expressing L1 3xFLAG tagged hFWE4, a shift from contiguous to punctate membrane signal was observed within 5 min, followed by perinuclear accumulation at 15 and 30 min (Fig. 6*A*). Given steady-state colocalization with early and recycling endosomes, we reasoned this shift in subcellular localization might represent clathrin-mediated endocytosis (CME) of surface-bound hFWE4 as was previously described for fly and mouse isoforms (5, 7). This hypothesis was tested with complementary imaging and biochemical approaches.

First, to determine whether inhibition of CME prevents hFWE4 relocalization following the onset of endocytosis, we transfected hFWE4 L1 3xFLAG expressing cells with a construct encoding a dominant negative dynamin1 K44A (Dyn1-K44A) mutant, which robustly inhibits dynamin-dependent vesicle scission during CME (16). Compared to control cells, Dyn1-K44A transfected cells better maintained membranous hFWE4 signal (Fig. 6*B*). Similarly, a mutant hFWE4 L1 3xFLAG construct harboring alanine repeats at the N-terminal (WWYRWL) and C-terminal (YARI) AP-2 binding motifs that had been previously suggested to promote internalization of mouse mFWE2, was properly trafficked and exposed at the cell surface, but exhibited no change in subcellular localization after 5 min of 37 °C chase (Fig. 6*C*). Lastly, to quantitatively assess the rate of hFWE4 internalization and the consequence of AP-2 motif recognition on this process, surface biotinylation and internalization assays on HEK293 cells expressing AP-2 wild-type or AP-2 mutant hFWE4 L1 3xFLAG constructs was performed. Immunoblotting for FLAG revealed rapid internalization of the AP-2 wild-type hFWE4 construct, with the internalized fraction exceeding 22% at 5 min, and reaching 71.6% (SEM ± 17.2%) within 30 min of endocytosis onset. Comparatively, only 26.2% (SEM ± 3.6%) of AP-2 mutant hFWE4 had been internalized within 30 min (*p* = 0.046) (Fig. 6*D*). The lack of tubulin signal in pulldowns confirmed the absence of cytosolic contamination. Together, these data strongly support a model in which hFWE4 exists transiently at the cell surface and is constitutively internalized in a clathrin-dependent manner that requires functional AP-2 binding motifs.

## Discussion

Flower isoforms appear to regulate a diverse set of biological processes, from fitness sensing during cell competition (1, 2, 3), to calcium channeling that drives tightly controlled endocytic events in a variety of cell types (4, 5, 6, 7). How Flower molecules direct these activities is unclear; however, several mechanistic hypotheses have been generated, each of which is reliant on specific isoform subcellular localizations and membrane topologies.

In *Drosophila*, Flower’s role as a “fitness fingerprint” is purported to rely on extracellular exposure of unique C-terminal tails generated through alternative splicing of exon 5. Interestingly, conflicting evidence regarding this structure exists in the literature. In their seminal work, Rhiner and colleagues utilized ectopic expression of N- or C-terminally HA-tagged Fwe-Ubi to demonstrate that in chemically fixed but unpermeabilized S2 cells, only C-terminal HA signal was detectable at the membrane (2). More recently, this was supported by extracellular labeling and acid-washing of fluorescent proteins fused to the C-terminus of *Drosophila* Fwe isoforms in unfixed wing disc whole mounts (10). While these data support a three TM domain model of Fwe that positions the C terminus in the extracellular space, they are in direct conflict with results from several sets of experiments performed by other groups. Chang and colleagues reported that an mRFP fused to the C-terminus of murine codon optimized Fwe-Ubi or its mouse ortholog, mFWE2, was only detectable with an α−mRFP antibody following membrane permeabilization of mouse cytotoxic T lymphocytes (CTLs) (7). Additionally, *in vitro* BRET experiments performed by Li and colleagues suggested that positively-charged residues at both the N- and C-terminus, as well as L2 of Fwe-LoseA, interact with cytosolic facing PI(4,5)P_2_ domains (6). These experiments both imply a four TM structure with cytosolic N- and C-terminal tails. The four TM pass model for mFWE2 was further supported in experiments that investigated cytosolic exposure of a pH-sensitive GFP (pHluorin) fused at the C-terminus or at L2 in live CTLs. Cytosolic acidification produced a significant ratiometric shift in the signal of pHluorin when fused to either domain, suggestive of cytoplasmic localization (7).

In our study, we utilized several complementary techniques that together provide strong evidence that the canonical human FWE isoform, hFWE4, also assumes this four TM structure with cytosolic termini. We showed that epitope tags appended to the N-terminus, C-terminus, or L2 were accessible at the cytosolic side of the membrane by genetically encoded Frankenbodies, while L1 and L3 were inaccessible to these cytosolic probes. Importantly, using these same expression constructs, surface staining revealed that only L1 and L3 were presented extracellularly in live cells. Given the lack of motif-specific hFWE antibodies, this epitope-tagging strategy is one of the few options to validate membrane structure and has been employed by other groups investigating the topology of FWE orthologs. As there is a possibility that the negatively charged FLAG epitope may perturb membrane structure, we further validated N- and C-terminal cytosolic exposure using FPP assays. Future work would be benefited from structural validation of untagged hFWE isoforms using minimally invasive strategies like the substituted cysteine accessibility method (SCAM) (17, 18). Despite this limitation, these data are in support of our structural predictions and demonstrate the utility of generating structural hypotheses for membrane proteins using AF2 modeling and subsequent MD simulations.

Additionally, our data here demonstrated that the non-canonical hFWE3 isoform was not trafficked to the plasma membrane and instead remained resident in the membrane of the ER. We have verified these distinct subcellular localizations in the human cutaneous squamous cell carcinoma cell line SCC13 (data not shown) to verify that this is not a phenomenon specific to HEK293 cells. These structural and subcellular localization data for human hFWE isoforms appear incompatible with a cell competition mechanism reliant on the extracellular display of isoform-specific C-terminal tails. In fact, given that extracellularly exposed loops of hFWE4 are short (4–12aa), it is difficult to envision a scenario where the hFWE4 molecule itself engages in receptor-ligand-type interactions with an adjacent cell. Instead, the mechanism may involve *cis*-interaction of hFWE4 with an effector molecule that then mediates cell-cell interactions or alternatively relies on hFWE4-regulated trafficking of cell surface proteins that drive these competitive interactions. This latter possibility is particularly intriguing given a well-conserved role for hFWE4 in clathrin-mediated endocytosis (4, 5, 6, 7).

Our data indicate that in human epithelia, hFWE4 was rapidly removed from the plasma membrane in a dynamin-1 and AP-2-dependent manner, strongly suggesting that previously reported roles for FWE isoforms in clathrin-mediated endocytosis are conserved in human. In fly and mouse, FWE appears to directly regulate clathrin-mediated endocytosis in a calcium-dependent manner to facilitate retrieval of cell type–specific cargo. In response to a strong stimulus at presynaptic terminal boutons, FWE-mediated calcium channeling is believed to initiate endocytosis that drives retrieval of exocytosed neurotransmitters (4, 5, 6). In mouse CTLs, Chang and colleagues have proposed that mFWE may act as a calcium-sensitive platform for recruiting adapter complex components to facilitate the endocytosis of cytotoxic granules (7). Our data raise interesting questions regarding the precise nature of hFWE4-containing vesicles in human epithelia, the cargo they may carry, and the role of calcium in regulating this process. In our work, only a small fraction of hFWE4 colocalized to Rab4, Rab5 or Rab11 positive endosomes and we were unable to identify a marker that accounts for the remainder of the vesicular hFWE4-EGFP signal. More work is required to precisely define the composition of these uncharacterized vesicles and identify any epithelia-specific cargo they may traffic to and from the membrane. We hypothesize that diversity in cargo may lead to cell-type-specific responses to hFWE4 expression and that this may manifest unique cellular behaviors, especially in cell types that are highly sensitive to alterations in intracellular calcium.

## Experimental procedures

### Structural prediction of Flower proteins

The primary structures of proteins were submitted to the AlphaFold2_mmseqs2 Jupyter notebook (https://colab.research.google.com/github/sokrypton/ColabFold/blob/mainAlphaFold2.ipynb) of the ColabFold server (11, 12). Detailed parameters for AF2 structural predictions and related ColabFold submissions can be found in the supplementary experimental procedures section.

### MD simulations

Two-step MD simulations were performed on rank one structures from ColabFold for hFWE3 and hFWE4. First, using the YASARA software package (19) the membrane-embedded structures or hFWE3 and hFWE4 were simulated for 500 ns using the AMBER ff14SB and LIPID14 force fields (20, 21). The lowest energy structures on the first two principal components (PCs) free energy landscape (FEL) for both proteins were submitted to the second set of MD simulations using the GROMACS package and the CHARMM36m (22) force field. Details of the MD simulations and trajectory analyses are described in the supplementary material. Lowest energy structure for both proteins on the FEL were determined and used as a structural guide for epitope tag insertions.

### Circular dichroism spectropolarimetry

Circular dichroism (CD) spectra of synthetic hFWE fragments were recorded by using a JASCO 810 spectropolarimeter (Tokyo, Japan). Peptides were dissolved in 25 mM phosphate buffer, 37.5 mM NaCl, pH 7.4, and the concentration of the stock solutions were determined by quantitative RP-HPLC analysis (23). Peptide solutions were diluted to 50 μM, except the concentration of Ac-FWE(51–57)-NH_2_ was 200 μM. Because of its hydrophobicity, Ac-hFWE4(121–142)-NH_2_ was dissolved in 33% ACN in water. CD spectra of Ac-hFWE(51–57)-NH_2_, Ac-FWE(84–95)-NH_2_, hFWE4(121–142)-NH_2_ and Ac-hFWE4(145–172) were also measured in the presence of 15%, 30% and 45% TFE in same buffer (v/v). The samples were loaded onto a 0.05-cm path length cell, and the temperature of the solutions was kept at 25 ^°^C by using a Jasco PFD-425 Peltier thermostated cell holder. The sensitivity was set to normal, and samples were scanned from 185 to 260 nm wavelengths at a scan speed of 100 nm/min with 0.1 nm data pitch. CD spectra were obtained by averaging 20 scans and subtracting the solvent baseline. To calculate secondary structure composition, each CD spectra was deconvoluted by the CDSSTR algorithm with the basis set 4 as reference using the DichroWeb CD analysis deconvolution server (24).

### Molecular cloning, plasmids, and cell lines

#### hFWE overexpression plasmids

pLenti CMV-MCS-EGFP-SV40-Puro (Addgene #73582) was used as the base vector for all hFWE-encoding lentiviral constructs generated in this study and cloning was performed using In-Fusion Snap Assembly mastermix (Takara Bio 638947). Inserts were synthesized as double-stranded gBlocks (IDT) with In-Fusion-compatible overhangs or generated *via* standard PCR-based amplification. For EGFP fusion constructs, EGFP was fused in-frame with the hFWE coding sequence at either the N- or C-terminus. For epitope-tagged hFWE3 constructs, a single hemagglutinin (HA) tag was fused in-frame with the hFWE3 coding sequence at either the N- or C-terminal tail or immediately following the codon for residue I58 (referred to as L1). For hFWE4, three tandem FLAG tags (3xFLAG) were fused in-frame with the hFWE4 coding sequence at either the N- or C-terminal tail or immediately following the codons for residues I53 (L1), N87 (L2) or L117 (L3). Epitope-tagged hFWEs were cloned in-frame with an upstream EGFP-P2A cassette to generate lentivectors that coexpress EGFP and hFWE.

#### Other plasmids

pLenti-Frankenbody-HA-mCherry (13) and pLenti-Frankenbody-FLAG-mRuby2 (14) were generated by In-Fusion cloning Frankenbody ORFs from CMV-15F11-HA-mCherry (Addgene #129591) and CMV-Frankenbody-FLAG-mRuby2 (Addgene #175294) into pLenti CMV MCS GFP SV40 Puro following removal of the EGFP coding sequence. 3xHA-LGR5-EGFP was provided by Joshua Snyder and generated as previously described (25). Plasmids encoding mCherry fused organellar markers; mCherry-ER-3, mCherry-Lysosomes-20, mCherry-Rab11a-7, mCherry-Rab4a-7, mCherry-Rab5a-7, mCherry-Rab7a-7, and mCherry-Golgi-7 (Addgene #55041, #55073, #55124, #55125, #55127 and #55052), were gifts from Michael Davidson. Second-generation lentiviral packaging vectors psPAX2 (Addgene #12260) and pMD2.G (Addgene #12259) were gifts from Didier Trono. K44A HA-dynamin1 pcDNA3.1 (Addgene #34683) was a gift from Sandra Schmid.

#### Cell lines

HEK293 cells (ATCC, CRL-1573) were maintained in DMEM-C, high-glucose DMEM supplemented with L-glutamine and sodium pyruvate (Gibco 1195065), 10% FBS (Sigma F0926) and 1% pen-strep (Gibco 15140122). HEK293FT cells (Invitrogen R70007) for lentiviral packaging were maintained in DMEM-C containing 500 μg/ml geneticin (Gibco 10131035). Cells were grown at 5% CO_2_ at 37 °C.

### Lentiviral packaging, transduction, and generation of stable HEK293 cells

Lentiviral particles were packaged in HEK293FT (Invitrogen) using psPAX2 and pMD2.G packaging vectors following standard techniques. Supernatants were used to infect HEK293 in suspension in 0.5 μg/ml polybrene and cells were subsequently puromycin selected to generate stable cell pools.

### Live cell imaging

#### Organelle colocalization

HEK293 cells stably expressing hFWE3-EGFP or hFWE4-EGFP were transfected with indicated mCherry constructs and seeded onto collagen-coated glass-bottom dishes for live confocal imaging. For labeling of the plasma membrane, untransfected hFWE3-EGFP or hFWE4-EGFP expressing HEK293 cells were incubated in 1× CellMask Deep Red prior to imaging. Imaging was performed on a Nikon TiE equipped with a Yokagawa Spinning disc using a Plan Apochromat Lambda 100× oil immersion objective lens. During live imaging, sample temperature and CO_2_ were maintained at 37 °C and 5%, respectively, in a Tokai Hit stage-top incubator.

#### Frankenbody imaging

HEK293 stably expressing EGFP only, or coexpressing non-fused EGFP and hFWE3 or hFWE4 harboring epitope tags at indicated positions, were transduced with lentivirus encoding HA or FLAG targeting Frankenbodies and imaged as described above. Where indicated, 1× CellMask DeepRed and 1 μg/ml Hoechst were used to label the plasma membrane and nucleus, respectively.

#### Fluorescence protease protection

Fluorescence protease protection assays were performed as originally described (15) with the following modifications: HEK293 cells expressing indicated constructs were washed 1× in KHM buffer (110 mM potassium acetate, 3 mM MgCl_2,_ 20 mM HEPES pH 7.2) and subsequently permeabilized with 80 μg/ml Digitonin (Promega) in KHM buffer for 90 s, followed by incubation with 50 μg/ml Proteinase K. Live confocal imaging was performed as described above, capturing 1-s intervals upon the addition of digitonin and continued for the remainder of the time course.

### Cell surface immunofluorescent labeling

#### Surface staining

HEK293 stably expressing epitope-tagged hFWE isoforms were subject to cell surface immunofluorescence as previously described (25). Briefly, cells were placed on ice and washed once with cold staining medium (SM: high glucose, HEPES, phenol red–free DMEM (Gibco 21063029) with 2% FBS). Cells were then incubated on ice for 45 min with rabbit polyclonal anti-FLAG (Sigma F7425) or anti-HA (CST C29F4) diluted 1:200 in cold SM. Where indicated, 1 μg/ml propidium iodide was included in the antibody cocktail to assess membrane permeability. Cells were then washed 4× in cold SM, fixed for 15 min in 4% methanol-free formaldehyde (Cell Signaling Technology 47746), then blocked and permeabilized with 5% normal goat serum, 1% bovine serum albumin and 0.12% Triton X-100 in PBS for 1 h before secondary labeling with goat anti-rabbit-647 (Invitrogen A2144) and counterstaining with Hoechst 33342.

#### Internalization assays

HEK293 stably expressing epitope-tagged hFWE4 constructs were transfected with pCDNA3.1 control vector or HA-K44A-dynamin1 pcDNA3.1 using Lipofectamine LTX/PLUS. Forty-eight h later, cells were pulsed with antibodies as described earlier for cell surface immunofluorescence using mouse anti-FLAG (Sigma F1804). Following washing, cells were chased at 37 °C for indicated timepoints before fixation, blocking, and permeabilization as described above. For internalization assays, surface labeled hFWE4 was detected using Alexa Fluor 647 Tyramide SuperBoost goat anti-mouse IgG reagents according to the manufacturer’s instructions (Invitrogen B40916). Following tyramide labeling, HA-tagged dynamin1 expressing cells were detected by incubating cells with rabbit anti-HA (Cell Signaling Technologies C29F4).

### Image processing and analysis

All post-acquisition image processing and analysis were performed in NIS-Elements AR (V5.21.03). For live cell imaging of mCherry transfected cells, full-thickness Z stacks were subject to 3D deconvolution. Maximum intensity projections of central Z slices, or single Z slices are presented where indicated.

### Surface biotinylation and internalization assays

HEK293 cells stably expressing epitope-tagged hFWE4 constructs were subject to surface biotinylation and internalization protocols adapted from previous work (26, 27, 28). Briefly, cells were incubated in 0.3 mg/ml EZ-Link-Sulfo-NHS-SS-Biotin (Thermo Fisher #21331) for 30 min on ice to biotinylate cell surface proteins. Unreacted biotin was subsequently quenched with 50 mM glycine. To assess the rate of internalization, following glycine quenching, cells were refed with warmed DMEM-C and incubated at 37 °C to allow internalization to occur for 5, 15 or 30 min. Cells were returned to ice, and the remaining surface biotin was stripped by incubating cells with cold 100 mM Mesna (Sigma #PHR1570) in 50 mM Tris pH8.6, 1 mM EDTA, and 100 mM NaCl for 20 min. Cells were then incubated with cold 50 mM iodoacetamide for 15 min on ice to quench residual Mesna and scraped into lysis buffer (1% Triton-X 100, 150 mM NaCl, 1 mM EDTA) supplemented with 1x HALT Protease and Phosphatase Inhibitor Cocktail (Thermo Fisher #78425). Protein estimation was performed to normalize protein quantity taken for input and used for pulldowns across samples. For pulldown of biotinylated protein, equal quantities of lysate were incubated with NeutrAvidin Agarose Resin (Thermo Fisher #29200) and rotated at 4 °C overnight. The resin was washed three times in lysis buffer and subsequently heated to 65 °C for 12 m in 4x Laemmli buffer to elute and denature biotinylated proteins prior to SDS-PAGE and immunoblotting. Primary antibodies used in immunoblotting included anti-FLAG (Sigma F1804), or anti-α/β Tubulin (Cell Signaling Technology #2148). The chemiluminescent signal was developed and quantified on a ChemiDoc XRS+ imaging system (Bio-Rad).

### Statistical analysis

Densitometric data were collected from ImageLab and transferred to GraphPad Prism for visualization and analysis. Statistical comparisons were made using two-way ANOVA with *post hoc* Holm-Sidak correction, exact *p* values are reported.

## Data availability

All data are contained within the presented article.

## Supporting information

This article contains supporting information.

## Conflict of interest

The authors declare that they have no conflicts of interest with the contents of this article.

## Supporting information

Supporting information

Supporting Figure S1 ***hFWE3* and *hFWE4* are the most abundant *hFWE* transcript species in human tissue.***A*, exon structure of *hFWE1-4* transcripts. *Black boxes* indicate 5’ and 3’ UTR. Exon 6a and 6b contain identical sequences, but inclusion of Exon 5 results in a +1 bp frameshift in the Exon 6 open reading frame to generate a unique coding sequence. *B*, Exon level expression for *hFWE* Exons 1-6 extracted from GTEx Analysis Release V8 representing median read count/base from surveyed human tissue types (N = 54). *C*, Isoform level expression for *hFWE 1-4* extracted from GTEx Analysis Release V8 representing median isoform TPM from surveyed human tissue types (N = 54). *D*, Isoform level expression data for *hFWE1-4* from long read Oxford nanopore technology-based cDNA sequencing of GTEx human tissue samples (N = 90) and K562 cell line samples (N = 4) (Glinos et al, 2022). Numerical annotations to the right of each violin plot indicate mean values.

Supporting Figure S2 **Tertiary structures of hFWE3 and hFWE4.***A*, *top*: Cartoon representation of the AlphaFold 2-predicted structure of hFWE3; *bottom*: PC1-PC2 free energy landscape of the YASARA MD simulation. Side bar represents the relative free energy in kJ mol^−1^. Cartoon representation of the lowest energy structure is indicated by an *arrow*. *B*, *top*: Cartoon representation of the AlphaFold 2-predicted structure of hFWE4; *bottom*: PC1-PC2 free energy landscape of the YASARA MD simulation. Side bar represents the relative free energy in kJ mol^−1^. Cartoon representation of the lowest energy structure is indicated by an *arrow*.

Supporting Figure S3 **Change in secondary structure during 500 ns MD simulation of the structure of hFWE3 using GROMACS.** Secondary structure content was determined using the defined secondary structure of proteins (DSSP) method. *A*, N-terminal tail (residues 1–18); *B*, loop 1 (residues 51–57); *C*, transmembrane 2 region (residues 121–142); *D*, C-terminal tail (residues 145–172). In hFWE3 sequences for TM2 and CT are identical to that of in hFWE4, therefore, for easier comparison with Fig S3, in *C* and *D* the residues are numbered as in hFWE4.

Supporting Figure S4 **Change in secondary structure during 1044 ns MD simulation of the structure of hFWE4 using GROMACS.** Secondary structure content was determined using the defined secondary structure of proteins (DSSP) method. *A*, N-terminal tail (residues 1–18); *B*, loop 1 (residues 51–57); *C*, loop 2 (residues 84–95); *D*, transmembrane 4 region (residues 121–142); *E*, C-terminal tail (residues 145–172).

Supporting Figure S5 **Free energy landscape of the PC1-PC2 subspace of the trajectory of 500 ns MD simulation of hFWE3 using GROMACS.** Side bar represent the relative free energy in kJ mol^−1^. The low energy conformations of each family was identified by partition around medoid clustering are shown in cartoon representation and *color* coding is the same as in Figure 2.

Supporting Figure S6 **Free energy landscape of the PC1-PC2 subspace of the trajectory of 1044 ns MD simulation of hFWE4 using GROMACS.** Side bar represent the relative free energy in kJ mol^−1^. The low energy conformations of each family was identified by partition around medoid clustering are shown in cartoon representation and *color* coding is the same as in Figure 2.

Supporting Figure S7 **The CD spectra of peptide fragments in different solutions.***A*, N-terminal tail (residues 1–18) in buffer; *B*, loop 1 (residues 51–57); *C*, loop 2 (residues 84–95); *D*, transmembrane 4 region (residues 121–142); *E*, C-terminal tail (residues 145–172). Buffer, *black*; 15% TFE, *blue*; 30% TFE, *red* and 45% TFE, *green*. In *panel D*, black represent 33% aqueous acetonitril.

Supporting Figure S8 **hFWE3 and hFWE4 do not colocalize with Rab7a positive endosomes, lysosomes or golgi.** HEK293 cells stably expressing (*A*) hFWE3-EGFP or (*B*) hFWE4-EGFP were transfected with indicated mCherry chimeras and live imaged for native EGFP and mCherry. Shown are single Z-slices from representative 100× live cell confocal images, scale bar = 10 μm.

Supporting Figure S9 **Cell surface immunofluorescent signal is not a product of membrane permeability.***A*, HEK293 stably coexpressing non-fused EGFP (*green*) and hFWE4-L1 3xFLAG were pulsed with anti-FLAG primary antibody on ice for 45 min in the presence of propidium iodide (PI) prior to fixation and secondary antibody labeling. *B*, as a positive control for PI incorporation and detection, HEK293 stably coexpressing non-fused EGFP (*green*) and hFWE4-L1 3xFLAG were fixed and permeabilized prior to incubation with propidium iodide. Shown are single Z-slices from representative 100× confocal images, scale bar = 10 μm.

Supporting Figure S10 **Frankenbodies demonstrate unique subcellular localizations of hFWE3 and hFWE4.** HEK293 cells stably expressing EGFP alone, or coexpressing non-fused EGFP and hFWE3 (*A*) or hFWE4 (*B*) harboring epitope tags at indicated positions were transduced with lentivirus encoding HA- or FLAG-targeting Frankenbodies. Cells were live imaged for native mCherry/mRuby2 fluorescence (*yellow*), CellMask Plasma Membrane label (*magenta*), and Hoechst (*blue*). Representative single Z-slices are presented from 100× live cell confocal images, scale bar = 10 μm. *C*, schematic overview of hFWE isoform structure on intracellular membranes, with hFWE4 assuming a four transmembrane pass structure and hFWE3 assuming a two transmembrane pass structure. Both isoforms position N and C termini cytosolically.
