# Supplementary material for: Membrane structure and internalization dynamics of human Flower isoforms hFWE3 and hFWE4 indicate a conserved endocytic role for hFWE4
Source: J Biol Chem. 2023 Jun 20;299(8):104945. doi: 10.1016/j.jbc.2023.104945 (PMC10366549; doi:10.1016/j.jbc.2023.104945)
Supplement: Supporting information [file mmc1.docx]

**SUPPORTING INFORMATION**

***SUPPORTING EXPERIMENTAL PROCEDURES***

***Structural prediction of Flower Proteins with ColabFold*.**

For submission of primary hFWE sequences to ColabFold, the following parameters were used: template mode, none; use_amber, yes; msa_mode, MMseqs2 (UniRef+environmental); pair_mode, unpaired+paired; model_type, auto; num_recycles, 5. Rank 1 for both proteins were used for further structural refinement using molecular dynamics (MD) simulations.

***MD Simulations***

First, Using the *md_runmembrane* macro of the YASARA software package (1), both proteins were embedded in a lipid membrane (2) which was composed of one hundred 1-palmytoil-2-oleyl-phophatidylcholine (POPC) phospholipid molecules on each sides and the membrane-protein system was solvated with water molecules and 150 mM NaCl. The simulations were run at 303 K and 1 atm pressure for 500 ns using the AMBER ff14SB and LIPID14 force field (2, 3). Trajectories were saved in xtc format and analyzed by the analysis utilities of the GROMACS (4) (version 2021.5) software package and in-house written R scripts. The sampled conformations of the proteins in the trajectories were analyzed using the Cartesian principal component ananlysis (PCA) of the C^α^ atoms. Eingenvaules and eigenvectors of covariance matrices were calculated by *gmx covar* and *gmx anaeig* was used to project trajectories on the eigenvectors of the covariance matrix to obtain the first two principal components (PCs). The two-dimensional (2D) free energy landscape (FEL) from the PC1-PC2 projection of the trajectory a three-dimesional (3D) histogram was created and the FEL was visualized, respectively, by *akima (5)* and *latticeExtra* (6) modules available in the R program. To identify the lowest energy structure on the FEL, the PC1-PC2 projection data was clustered and visualized by the partion around medoid (PAM) method of the *cluster* (7) and *factoextra* (8) modules, respectively, of the R software.

Second, The lowest energy structures of the proteins were then submitted to another MD simulation using the GROMACS package and the CHARMM36m (9) force field. For the simulations, the proteins were embedded in POPC lipid membrane and solvated by TIP3P water molecules using the CHARMM-GUI Membrane Builder (10). The lipid membrane for hFWE3 consisted of 114 lipid molecules on each sides and it was solvated by 26,411 water molecules and 150 mM NaCl. The lipid membrane for hFWE4 consisted of 200 lipid molecules on each sides and it was solvated by 41,947 water molecules and 150 mM NaCl. Systems were energy-minimized and equilibrated using the default CHARMM-GUI Membrane Builder equilibration protocol. Production NPT ( 303.15 K and 1 atm pressure) simulations were 500 ns and 1044 ns for hFWE3 and hFWE4, respectively. Trajectories were integrated with 2 fs time step. Heavy atom-hydrogen bonds were constrained to their correct length using LINCS, with a warning angle of 30 degrees (11). The long-range electrostatic interactions were calculated using the PME method with 1.2 nm cutoff distance and 0.12 nm Fourier spacing (12). Van der Waals interactions were calculated using short-range and long-range cutoffs of 1.0 and 1.2 nm, respectively, with a force-switch modifier (13). The temperature was maintained by the Nosé-Hoover method (14); Protein, membrane and solvent were indpendently coupled to the temperature bath with 1 ps coupling constant. The system was coupled to a Parrinello-Rahman semiisotropic barostat (15) with a time constant of 5 ps and compressibility of 4.5 10^-5^ bar^-1^.

***Materials***

N^α^-Fmoc-protected amino acids, piperidine, diethyl ether, Triisopropylsilane (TIS), Trifluoro acetic acid (TFA), N,N’-Diisopropylcarbodiimide (DIC), Rink amide resin, DMF, HPLC grade acetonitrile (ACN) and TFA and 2,2,2-trifluoroethanol (TFE) were purchased Sigma-Aldrich (St. Louis, MO). Oxymapure was purchased from CEM corporation (Matthews, NC).

***Peptide synthesis and purification****.* The peptide fragments of hFWE were either purchased or synthesized in our laboratory. For intrachain fragments, *N*-acetyl and amide protecting groups were added to the N- and C-terminus, respectively, in order to preserve the electronic structure of the backbone as in hFWE. Ac-hFWE(51-57)-NH_2_ (L1), Ac-hFWE4(84-95)-NH_2_ (L2) and Ac-hFWE4(145-172) (CT) were purchased from vivitide (Gargner, MA; Vivitide now re-branded as BioSynth); Ac-hFWE4(121-142)-NH_2_ (TM4)) was purchased from Peptide 2.0 (Chantilly, VA). Purity of the peptides were grater than 95% as determined by reverse phase high-performance liquid chromatography (RP-HPLC). hFWE(1-18)-NH_2_ (NT) was synthesized using solid phase peptide synthesis (SPPS) method at 0.1 mmol scale using the CEM Liberty Microwave peptide synthesizer. Rink amide resin with 0.78 mmol/g loading capacity was used as solid support. A 5 molar excess of N^α^-Fmoc-protected amino acids were dissolved in DMF at 0.2 M concentration. 0.5 M DIC : 0.5 M Oxymapure (1:1) in DMF was used as coupling reagent. Coupling reactions were at 90 ^O^C for 2 min. N^α^-Fmoc removal was performed using 10% (v/v) piperidine in DMF at 90 ^O^C for 2 min. The peptide was cleaved from the resin using a mixture of TFA:TIS:H_2_O (95:2.5:2.5, v/v/v). The peptide-resin and a cleavage mixture was stirred in a round bottom flask at 0 ^o^C for 30 min, and then at room temperature for 90 min. The peptide was then precipitated with ice-cold diethyl ether, separated by filtration, dissolved in 10% AcOH in H_2_O and lyophilized. The crude peptide was purified with semi-preparative RP-HPLC (Gilson, Middleton, USA) using Phenomenex Luna C18, 5µm, 250 x 10 mm column, solvent A: 0.09% TFA in water, solvent B: 0.1% TFA in ACN, flow rate 4 mL/min, gradient 5-60 % solvent B in 60 min. Purity of the peptide was detremined by analytical RP-HPLC (column: Phenomenex Jupiter C18, 10 µm, 250 x 4.6 mm; solvent A: 0.09% TFA in water, solvent B: 0.1% TFA in ACN, flow rate 1 mL/min, gradient 5-60 % solvent B in 30 min) and the identity of was confirmed by MALDI-Tof mass spectrometry. Expected mass: 1488.58 Da, observed 1488.752 Da.

***SUPPORTING RESULTS***

***MD Simulations***

In the embedding process of hFWE3 the two tails and the loop (L) that connects the two-helix bundle are positioned on the opposite side of the membrane and the two helices formed transmembrane (TM) locations. PCA of the 500 ns trajectory of the hFWE3 MD simulation resulted in a free energy landscape (FEL) that has one broad minimum with the lowest energy and several small local minima (Fig S2A, middle). In the lowest energy structure on the FEL (Fig S2A, bottom) the N- and C-tails are more tilted than in the AF2-predicted structure of hFWE3. The structure of hFWE4 was embedded in such way that the four-helix bundle formed a TM structure, N- and C-tails are on the same side of the membrane and Loop 1 and 3 (L1 and L3) are on the opposite side. The short helical loop 2 (L2) is located on the N- and C-tail side of the membrane surface. PCA of the 500 ns trajectory of the hFWE4 MD simulation resulted in a FEL that has one broad and deep minimum (Fig S2B, middle). In the lowest energy structure on the FEL (Fig S2B, bottom) the overall 3D structure of hFWE4 is similar to that predicted by AF2 with the exception of the N-tail that tends to be more folded up, but still in random meander conformation (Fig S2B).

In a benchmark study of various lipid forcefields, it was shown that the CHARMM36 force field reproduces better various lipid membrane properties than does the LIPID14 force field (16). Therefore, to further refine the predicted 3D structures of the proteins, the lowest energy structures on the FELs of YASARA simulations for both proteins were submitted to subsequent MD simulation using the GROMACS software package and the CHARMM36 force field. During 500 ns MD simulation of hFWE3, the initial secondary structure of the N-tail and TM helix 2 (Fig. S3 A and C) did not substantially change. Whereas the secondary structure of the single loop motif (L) during the initial membrane embedding and energy minimization converted from helix to mostly β-turn/bend structures and stayed in such structure (Fig. S3B). The helix of the C-terminal tail, on the other hand, was not stable and broke into shorter helix and mostly random coil structure (Fig S3D). During the 1044 ns MD simulation of hFWE4, all the structural motifs that are shared with hFWE3 followed similar secondary structural changes (Fig. S3 A,B, D and E). The membrane surface L2 formed a stable helix (Fig. S4C). PCA of both simulations resulted in more rugged FELs than for the simulations with AMBER ff14SB/LIPID14 force fields indicating that the proteins explored several local minima (Fig S5 and 6).

***CD Spectropolarimetry Analysis***

As we did for another membrane-associated protein characterization (17), for regions that are inside the membrane or in close contact with it, we used increasing concentrations of TFE to measure the CD spectra of the peptide fragments. The peptide fragments are out of the surrounding protein and membrane environment. Therefore, it is possible that they will not assume the same secondary structure in aqueous solution as in the protein. Subsequently, we followed the well-accepted practice of measuring CD spectra of the peptide in structure-promoting TFE solutions as well (18). Because both NT and CT peptides are fully outside of the membrane and their CD spectrum was only measured in buffer (Table 1 and Fig S7A and C). In agreement with simulation data the secondary structure of the NT peptide was a mixture of helix, turns, sheet and unordered conformation. The CT peptide, on the other hand, has a more pronounced helical conformation with a mixture of sheet and unordered conformations (Figs S6C, S3D and S4E). As expected, the transmembrane fragment TM4 was hydrophobic, and we were able to dissolve it in 33% aqueous ACN. CD spectra of L1 peptide in increasing TFE concentrations indicated a stabile β-turn/bend conformations. L2, which is laying on the membrane surface, had a strong tendency to form helical conformation in increasing TFE concentrations (Table 1 and Fig 7S). TM4 peptide is already mostly in helical conformation in 33% aqueous ACN (Table 1 and fig S6D) and it had the highest helicity content in the presence of 30 TFE.

***SUPPORTING INFORMATION REFERENCES***

1. Krieger, E., and Vriend, G. (2015) New ways to boost molecular dynamics simulations. *J Comput Chem*. **36**, 996–1007

2. Dickson, C. J., Madej, B. D., Skjevik, Å. A., Betz, R. M., Teigen, K., Gould, I. R., and Walker, R. C. (2014) Lipid14: The Amber Lipid Force Field. *J. Chem. Theory Comput.* **10**, 865–879

3. Maier, J. A., Martinez, C., Kasavajhala, K., Wickstrom, L., Hauser, K. E., and Simmerling, C. (2015) ff14SB: Improving the Accuracy of Protein Side Chain and Backbone Parameters from ff99SB. *J. Chem. Theory Comput.* **11**, 3696–3713

4. Abraham, M. J., Murtola, T., Schulz, R., Páll, S., Smith, J. C., Hess, B., and Lindahl, E. (2015) GROMACS: High performance molecular simulations through multi-level parallelism from laptops to supercomputers. *SoftwareX*. **1–2**, 19–25

5. code (TOMS 760, H. A. (Fortran, 761, and 433)), 697, port (interp*, A. G. (R, functions), bicubic*, code), bilinear, function), T. P. (aspline, enhancements), M. M. (interp2xyz function +, Machinery, Y. A. for C., TOMS 760, I. (covers code from, 761, and and 433), 697 (2022) akima: Interpolation of Irregularly and Regularly Spaced Data. [online] https://CRAN.R-project.org/package=akima (Accessed January 23, 2023)

6. Sarkar, D., and Andrews, F. (2022) latticeExtra: Extra Graphical Utilities Based on Lattice. [online] https://CRAN.R-project.org/package=latticeExtra (Accessed January 23, 2023)

7. Maechler, M., original), P. R. (Fortran, original), A. S. (S, original), M. H. (S, Hornik [trl, K., maintenance(1999-2000)), ctb] (port to R., Studer, M., Roudier, P., Gonzalez, J., Kozlowski, K., pam()), E. S. (fastpam options for, and Murphy (volume.ellipsoid({d >= 3})), K. (2022) cluster: “Finding Groups in Data”: Cluster Analysis Extended Rousseeuw et al. [online] https://CRAN.R-project.org/package=cluster (Accessed January 23, 2023)

8. Kassambara, A., and Mundt, F. (2020) factoextra: Extract and Visualize the Results of Multivariate Data Analyses. [online] https://CRAN.R-project.org/package=factoextra (Accessed January 23, 2023)

9. Huang, J., Rauscher, S., Nawrocki, G., Ran, T., Feig, M., de Groot, B. L., Grubmüller, H., and MacKerell, A. D. (2017) CHARMM36m: an improved force field for folded and intrinsically disordered proteins. *Nat Methods*. **14**, 71–73

10. Lee, J., Patel, D. S., Ståhle, J., Park, S.-J., Kern, N. R., Kim, S., Lee, J., Cheng, X., Valvano, M. A., Holst, O., Knirel, Y. A., Qi, Y., Jo, S., Klauda, J. B., Widmalm, G., and Im, W. (2019) CHARMM-GUI Membrane Builder for Complex Biological Membrane Simulations with Glycolipids and Lipoglycans. *J. Chem. Theory Comput.* **15**, 775–786

11. Hess, B., Bekker, H., Berendsen, H. J. C., and Fraaije, J. G. E. M. (1997) LINCS: A linear constraint solver for molecular simulations. *Journal of Computational Chemistry*. **18**, 1463–1472

12. Essmann, U., Perera, L., Berkowitz, M. L., Darden, T., Lee, H., and Pedersen, L. G. (1995) A smooth particle mesh Ewald method. *J. Chem. Phys.* **103**, 8577–8593

13. Lindahl, Abraham, Hess, and Spoel, V. D. (2021) GROMACS 2021 Manual. 10.5281/ZENODO.4457591

14. Martyna, G. J., Klein, M. L., and Tuckerman, M. (1992) Nosé–Hoover chains: The canonical ensemble via continuous dynamics. *J. Chem. Phys.* **97**, 2635–2643

15. Parrinello, M., and Rahman, A. (1981) Polymorphic transitions in single crystals: A new molecular dynamics method. *Journal of Applied Physics*. **52**, 7182–7190

16. Pluhackova, K., Kirsch, S. A., Han, J., Sun, L., Jiang, Z., Unruh, T., and Böckmann, R. A. (2016) A Critical Comparison of Biomembrane Force Fields: Structure and Dynamics of Model DMPC, POPC, and POPE Bilayers. *J. Phys. Chem. B*. **120**, 3888–3903

17. Lovas, S., He, D. Z. Z., Liu, H., Tang, J., Pecka, J. L., Hatfield, M. P. D., and Beisel, K. W. (2015) Glutamate transporter homolog-based model predicts that anion-π interaction is the mechanism for the voltage-dependent response of prestin. *J Biol Chem*. **290**, 24326–24339

18. Naider, F. (2007) Synthesis, biosynthesis, and characterization of transmembrane domains of a G protein-coupled receptor. *Methods Mol Biol*. **386**, 95–121
