# Supplementary figures and images for: Membrane structure and internalization dynamics of human Flower isoforms hFWE3 and hFWE4 indicate a conserved endocytic role for hFWE4
Source: J Biol Chem. 2023 Jun 20;299(8):104945. doi: 10.1016/j.jbc.2023.104945 (PMC10366549; doi:10.1016/j.jbc.2023.104945)

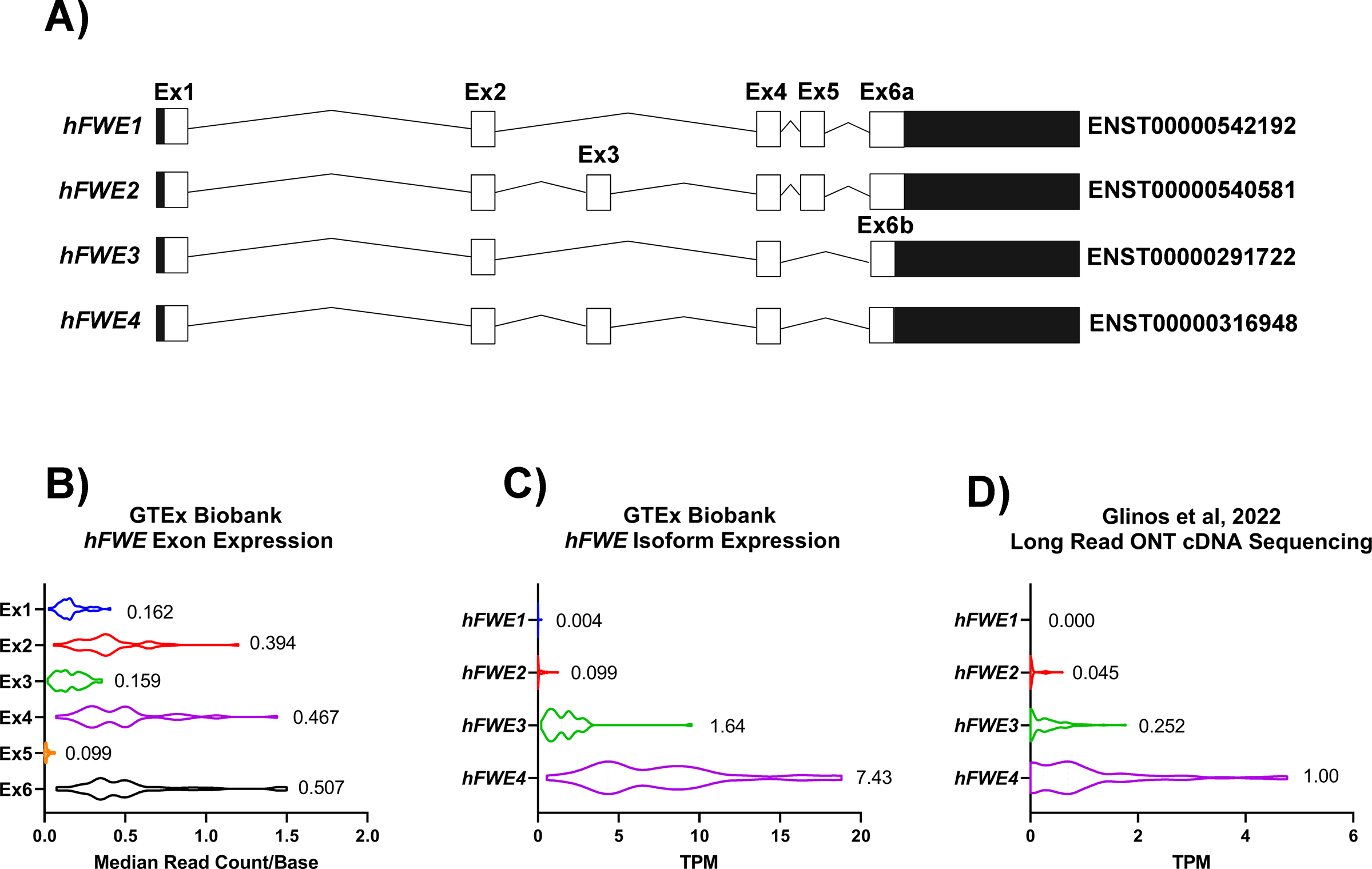

Supplement: Supporting Figure S1 — hFWE3 and hFWE4 are the most abundant hFWE transcript species in human tissue.A, exon structure of hFWE1-4 transcripts. Black boxes indicate 5’ and 3’ UTR. Exon 6a and 6b contain identical sequences, but inclusion of Exon 5 results in a +1 bp frameshift in the Exon 6 open reading frame to generate a unique coding sequence. B, Exon level expression for hFWE Exons 1-6 extracted from GTEx Analysis Release V8 representing median read count/base from surveyed human tissue types (N = 54). C, Isoform level expression for hFWE 1-4 extracted from GTEx Analysis Release V8 representing median isoform TPM from surveyed human tissue types (N = 54). D, Isoform level expression data for hFWE1-4 from long read Oxford nanopore technology-based cDNA sequencing of GTEx human tissue samples (N = 90) and K562 cell line samples (N = 4) (Glinos et al, 2022). Numerical annotations to the right of each violin plot indicate mean values. [file figs1.jpg]

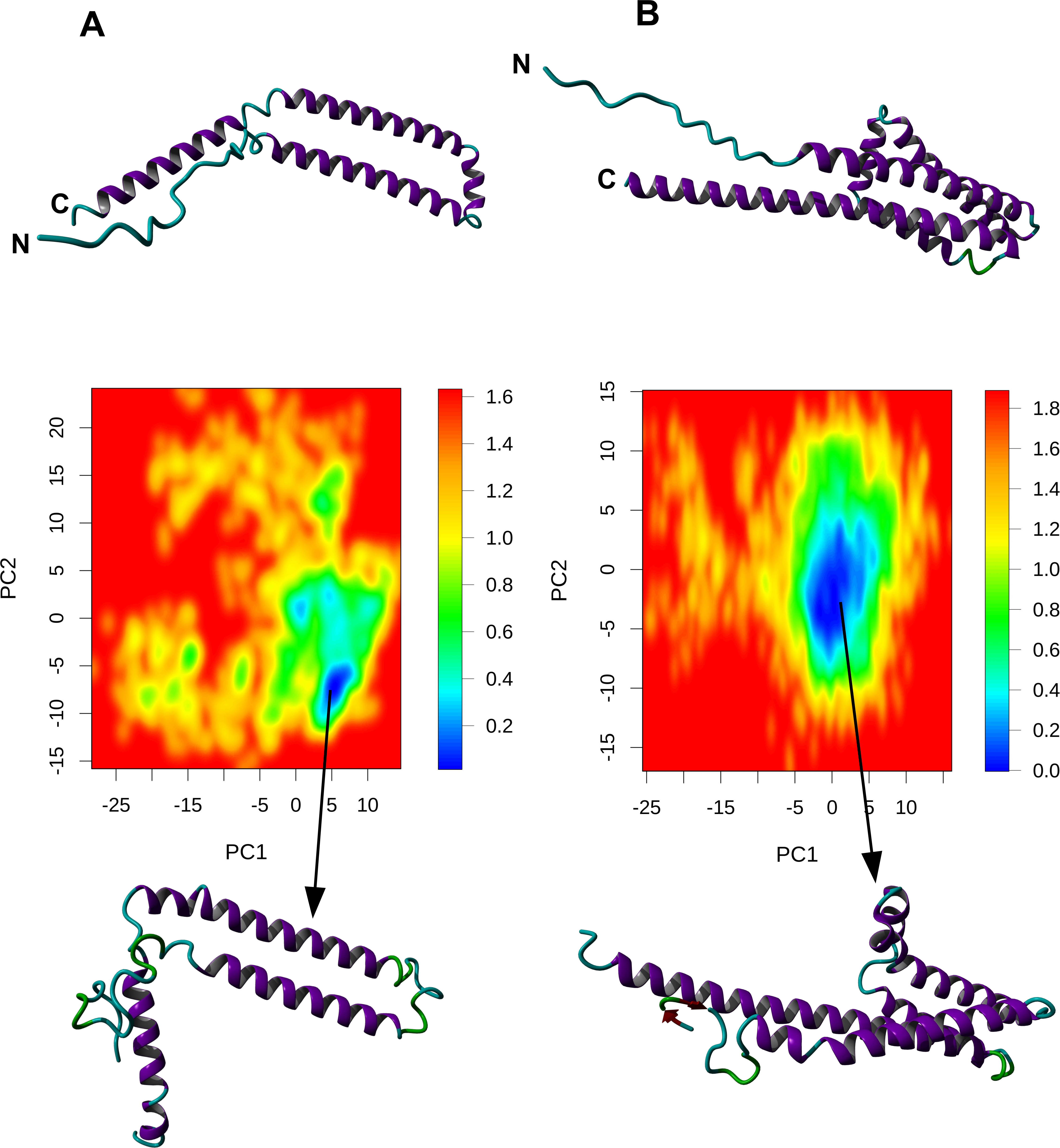

Supplement: Supporting Figure S2 — Tertiary structures of hFWE3 and hFWE4.A, top: Cartoon representation of the AlphaFold 2-predicted structure of hFWE3; bottom: PC1-PC2 free energy landscape of the YASARA MD simulation. Side bar represents the relative free energy in kJ mol−1. Cartoon representation of the lowest energy structure is indicated by an arrow. B, top: Cartoon representation of the AlphaFold 2-predicted structure of hFWE4; bottom: PC1-PC2 free energy landscape of the YASARA MD simulation. Side bar represents the relative free energy in kJ mol−1. Cartoon representation of the lowest energy structure is indicated by an arrow. [file figs2.jpg]

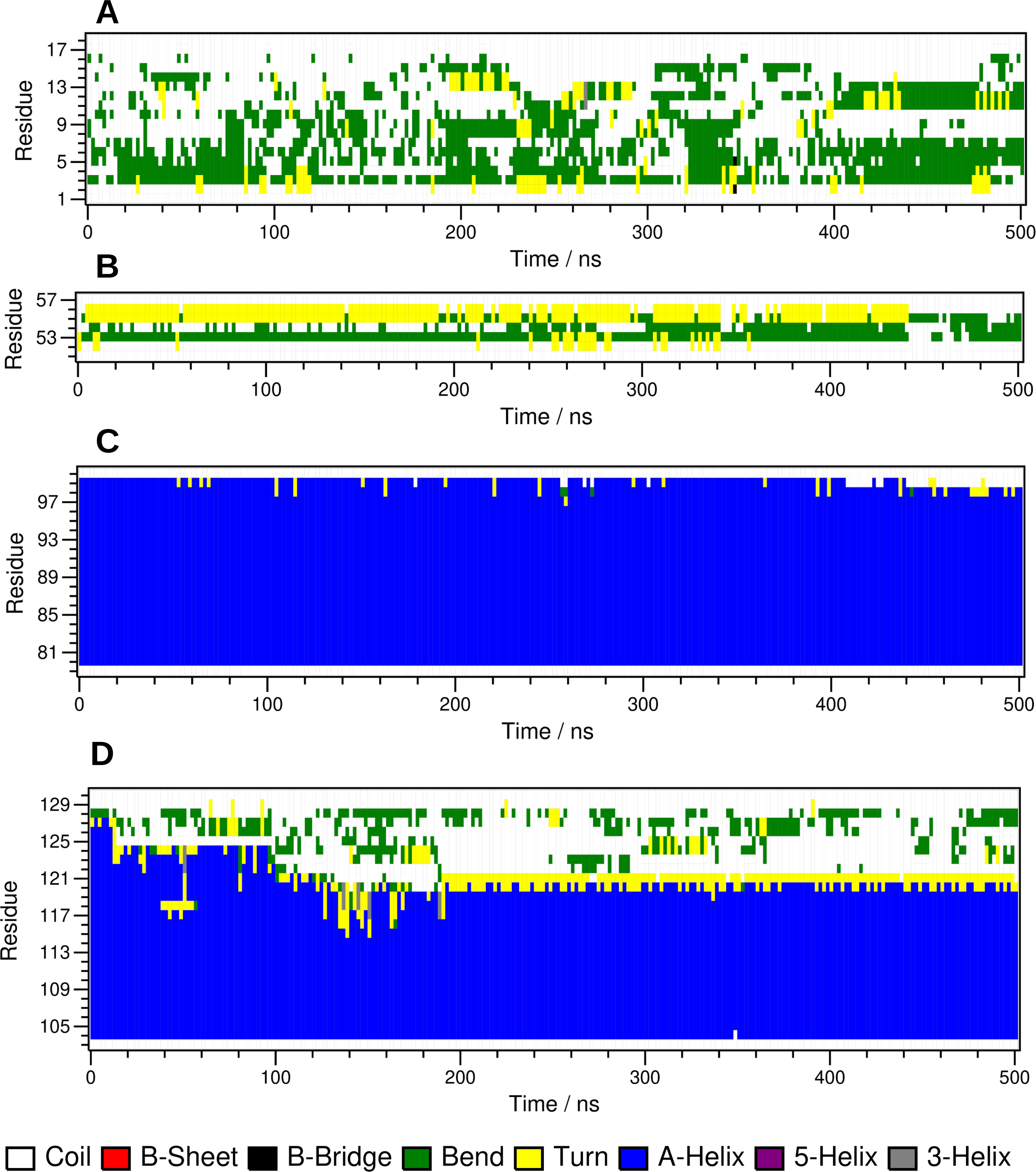

Supplement: Supporting Figure S3 — Change in secondary structure during 500 ns MD simulation of the structure of hFWE3 using GROMACS. Secondary structure content was determined using the defined secondary structure of proteins (DSSP) method. A, N-terminal tail (residues 1–18); B, loop 1 (residues 51–57); C, transmembrane 2 region (residues 121–142); D, C-terminal tail (residues 145–172). In hFWE3 sequences for TM2 and CT are identical to that of in hFWE4, therefore, for easier comparison with Fig S3, in C and D the residues are numbered as in hFWE4. [file figs3.jpg]

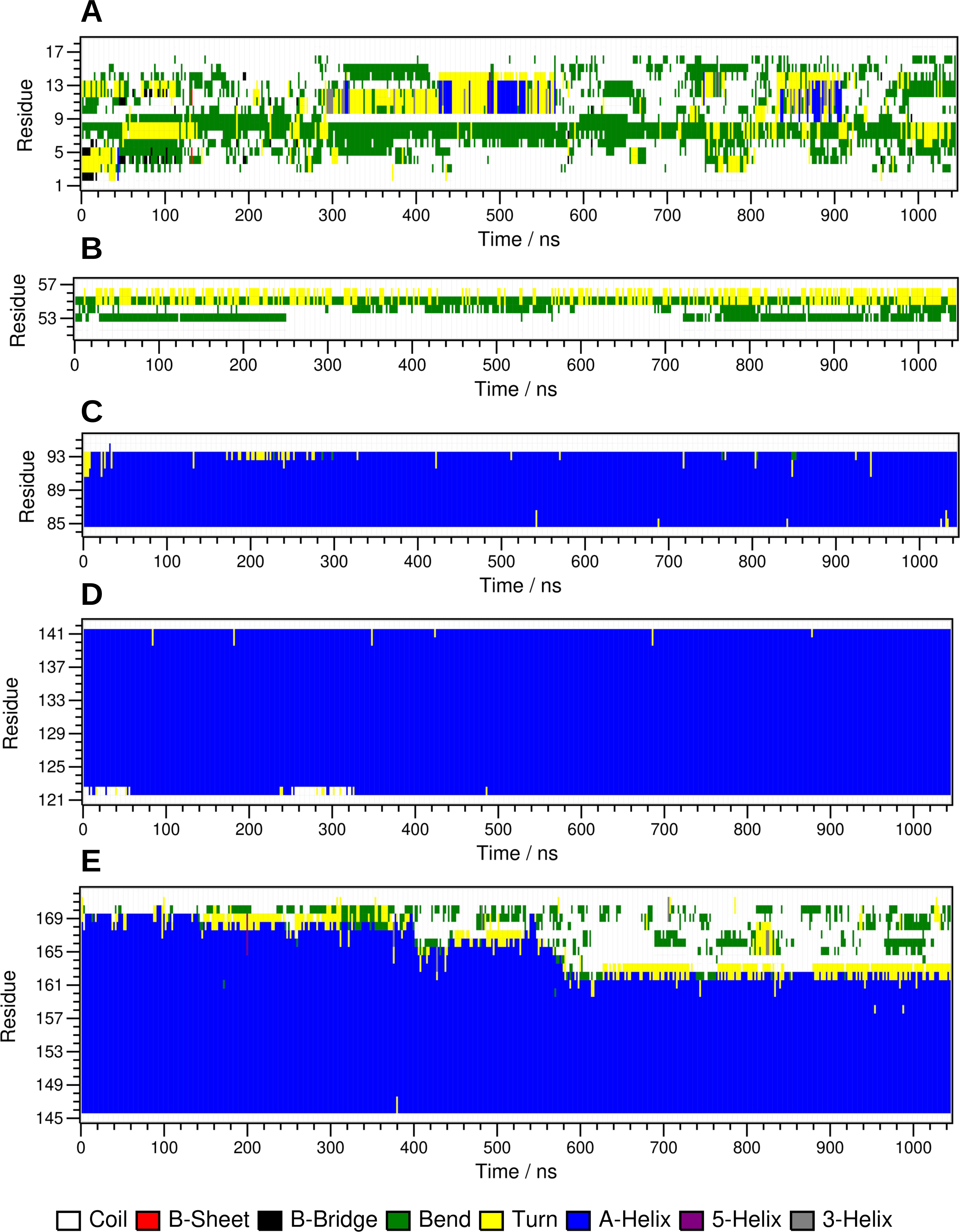

Supplement: Supporting Figure S4 — Change in secondary structure during 1044 ns MD simulation of the structure of hFWE4 using GROMACS. Secondary structure content was determined using the defined secondary structure of proteins (DSSP) method. A, N-terminal tail (residues 1–18); B, loop 1 (residues 51–57); C, loop 2 (residues 84–95); D, transmembrane 4 region (residues 121–142); E, C-terminal tail (residues 145–172). [file figs4.jpg]

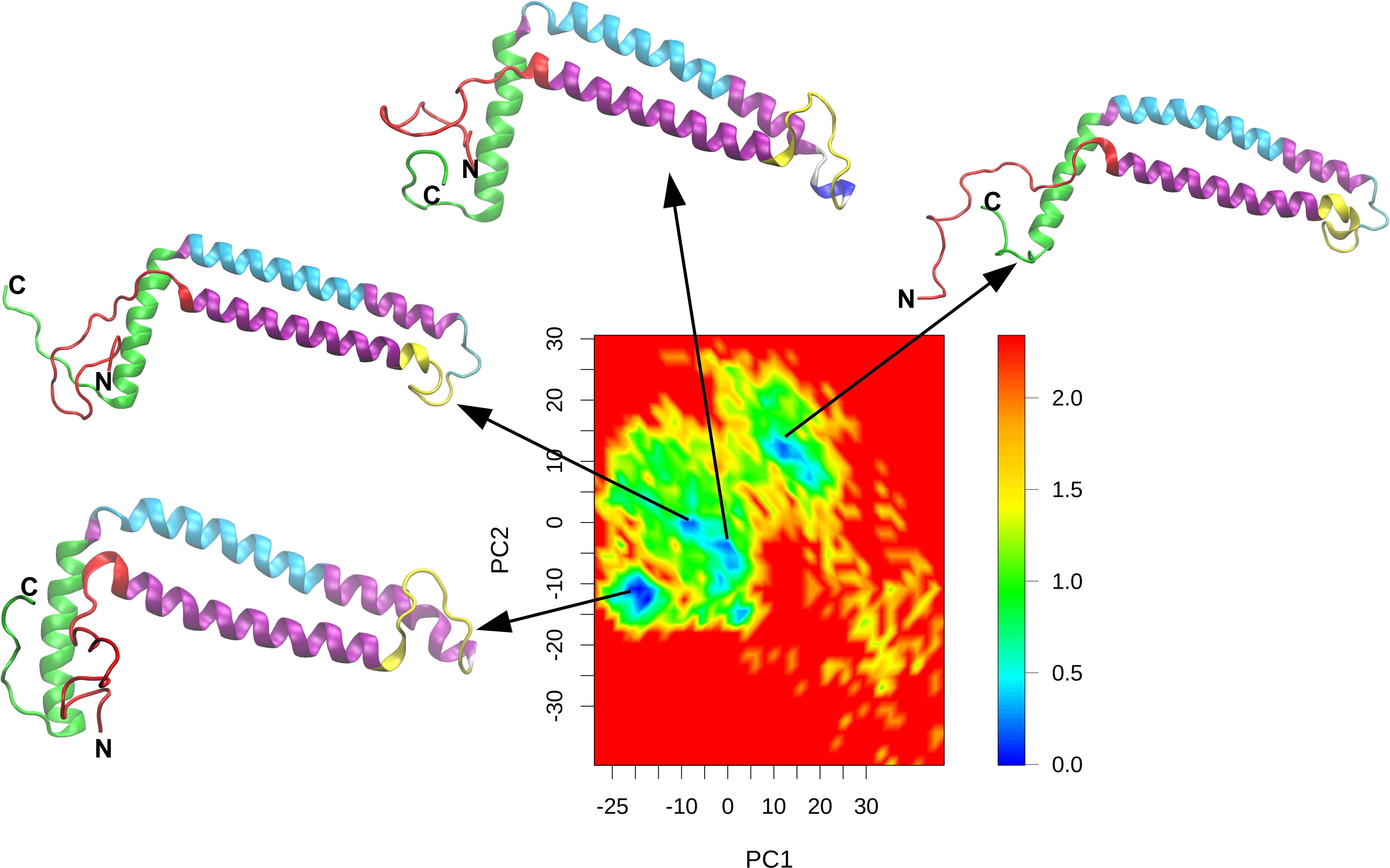

Supplement: Supporting Figure S5 — Free energy landscape of the PC1-PC2 subspace of the trajectory of 500 ns MD simulation of hFWE3 using GROMACS. Side bar represent the relative free energy in kJ mol−1. The low energy conformations of each family was identified by partition around medoid clustering are shown in cartoon representation and color coding is the same as in Figure 2. [file figs5.jpg]

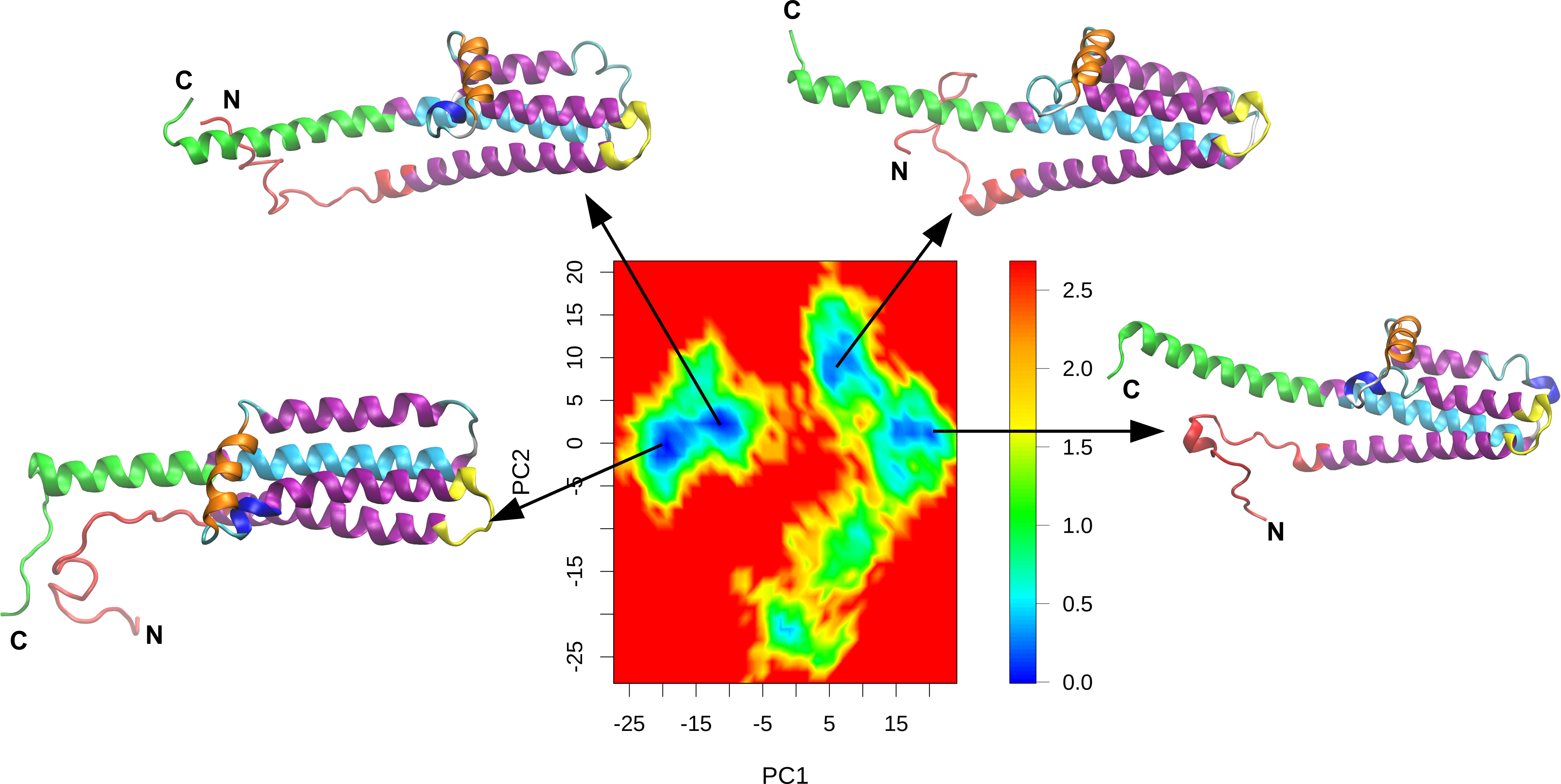

Supplement: Supporting Figure S6 — Free energy landscape of the PC1-PC2 subspace of the trajectory of 1044 ns MD simulation of hFWE4 using GROMACS. Side bar represent the relative free energy in kJ mol−1. The low energy conformations of each family was identified by partition around medoid clustering are shown in cartoon representation and color coding is the same as in Figure 2. [file figs6.jpg]

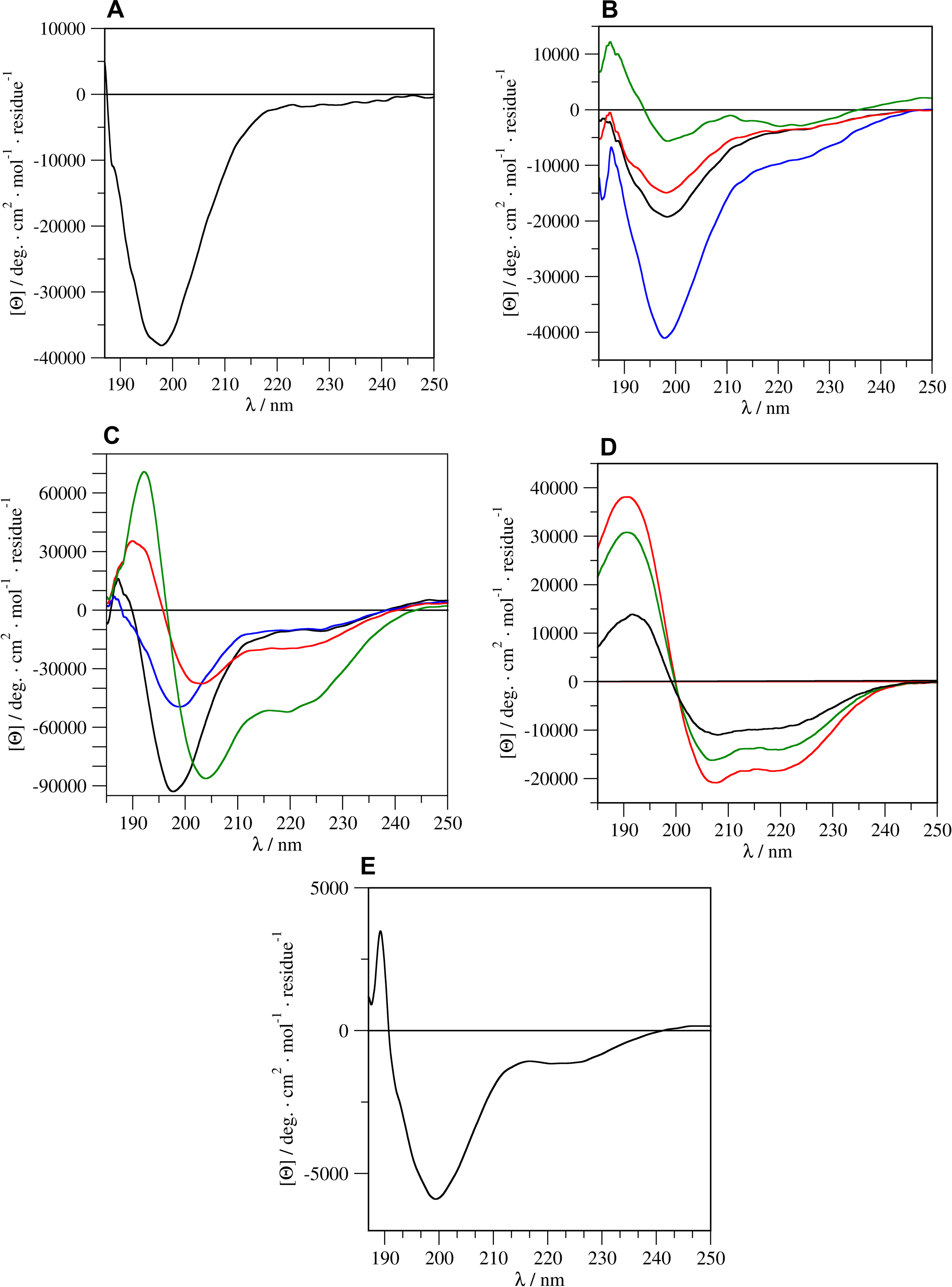

Supplement: Supporting Figure S7 — The CD spectra of peptide fragments in different solutions.A, N-terminal tail (residues 1–18) in buffer; B, loop 1 (residues 51–57); C, loop 2 (residues 84–95); D, transmembrane 4 region (residues 121–142); E, C-terminal tail (residues 145–172). Buffer, black; 15% TFE, blue; 30% TFE, red and 45% TFE, green. In panel D, black represent 33% aqueous acetonitril. [file figs7.jpg]

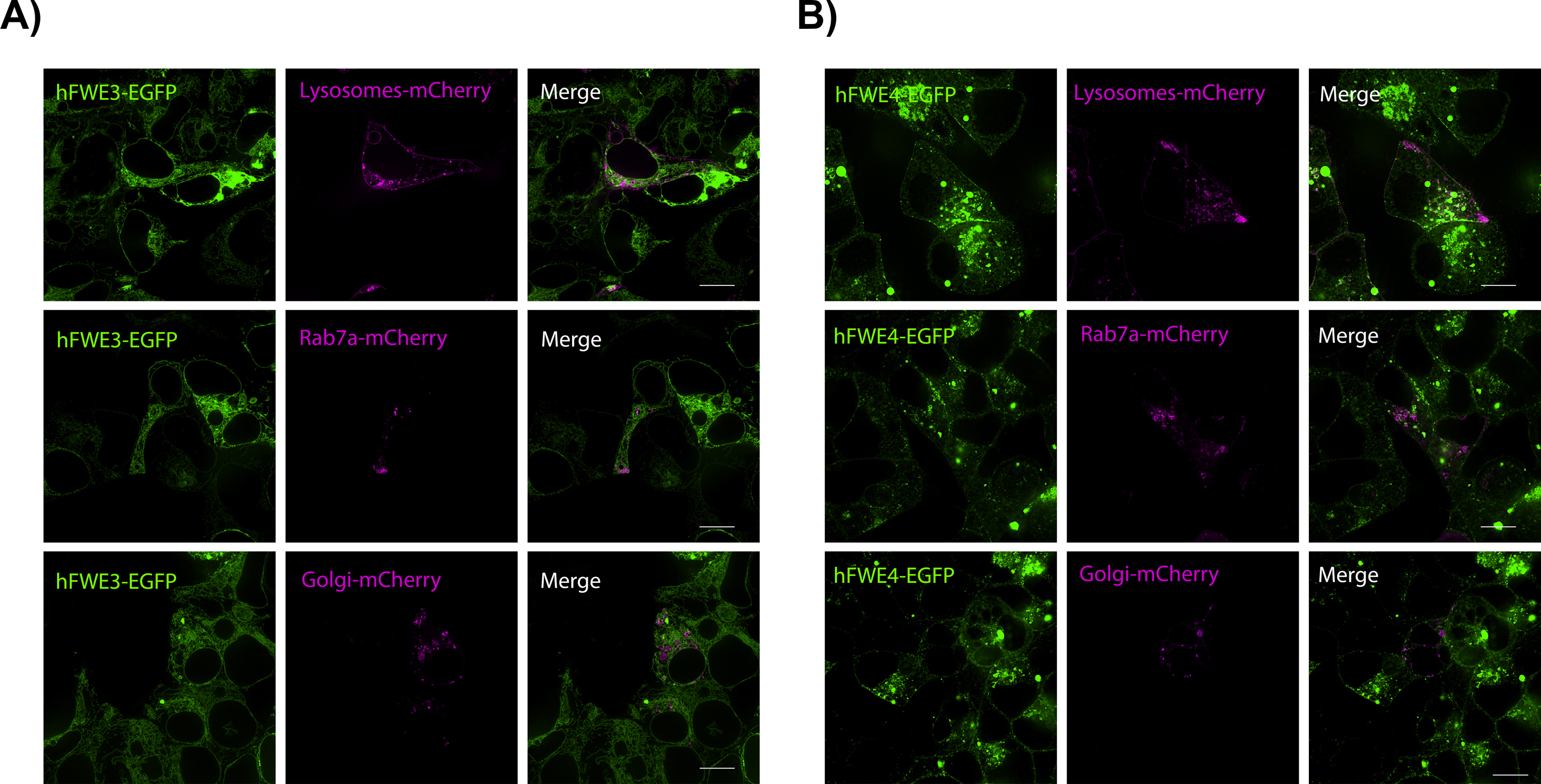

Supplement: Supporting Figure S8 — hFWE3 and hFWE4 do not colocalize with Rab7a positive endosomes, lysosomes or golgi. HEK293 cells stably expressing (A) hFWE3-EGFP or (B) hFWE4-EGFP were transfected with indicated mCherry chimeras and live imaged for native EGFP and mCherry. Shown are single Z-slices from representative 100× live cell confocal images, scale bar = 10 μm. [file figs8.jpg]

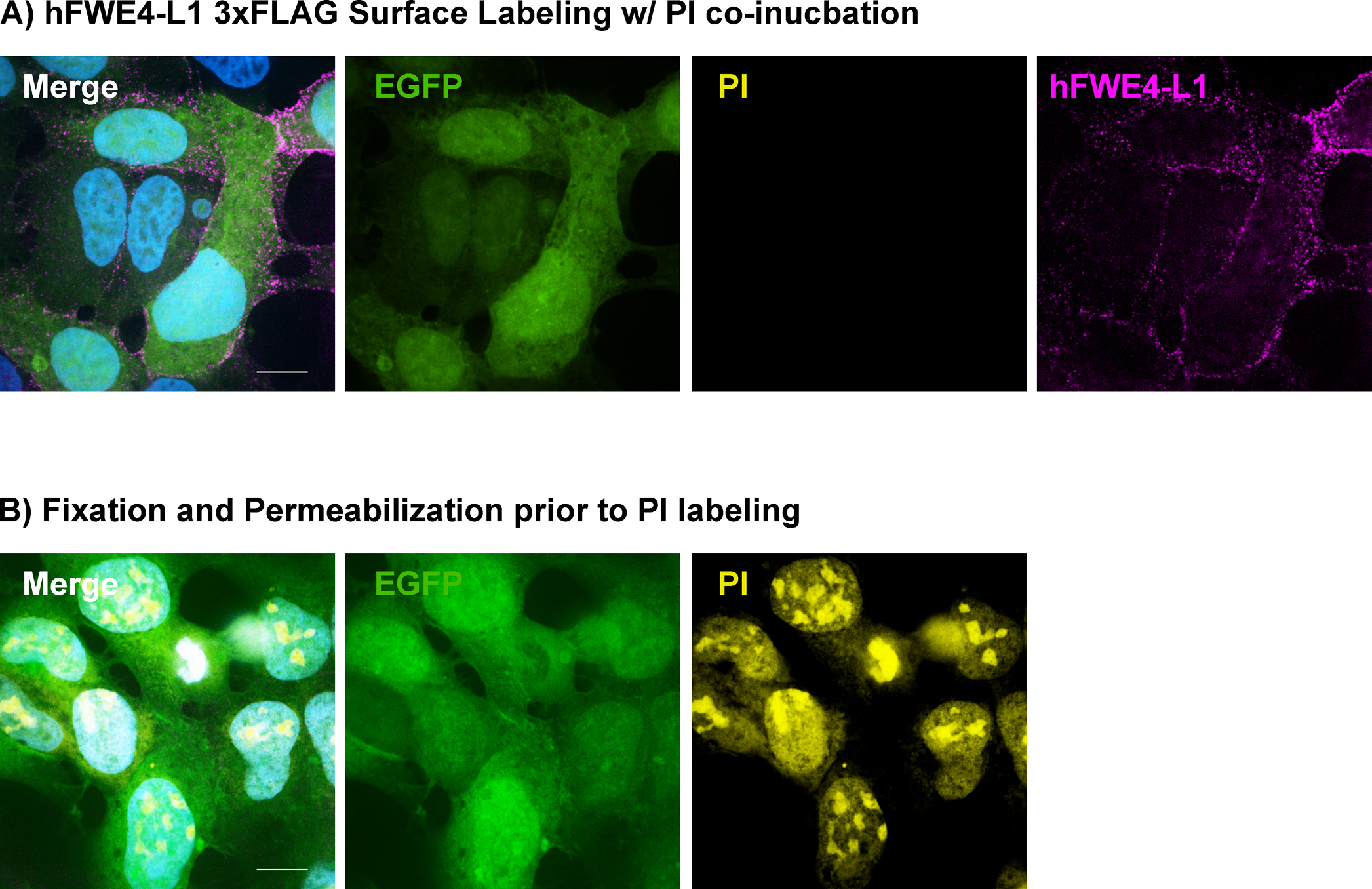

Supplement: Supporting Figure S9 — Cell surface immunofluorescent signal is not a product of membrane permeability.A, HEK293 stably coexpressing non-fused EGFP (green) and hFWE4-L1 3xFLAG were pulsed with anti-FLAG primary antibody on ice for 45 min in the presence of propidium iodide (PI) prior to fixation and secondary antibody labeling. B, as a positive control for PI incorporation and detection, HEK293 stably coexpressing non-fused EGFP (green) and hFWE4-L1 3xFLAG were fixed and permeabilized prior to incubation with propidium iodide. Shown are single Z-slices from representative 100× confocal images, scale bar = 10 μm. [file figs9.jpg]

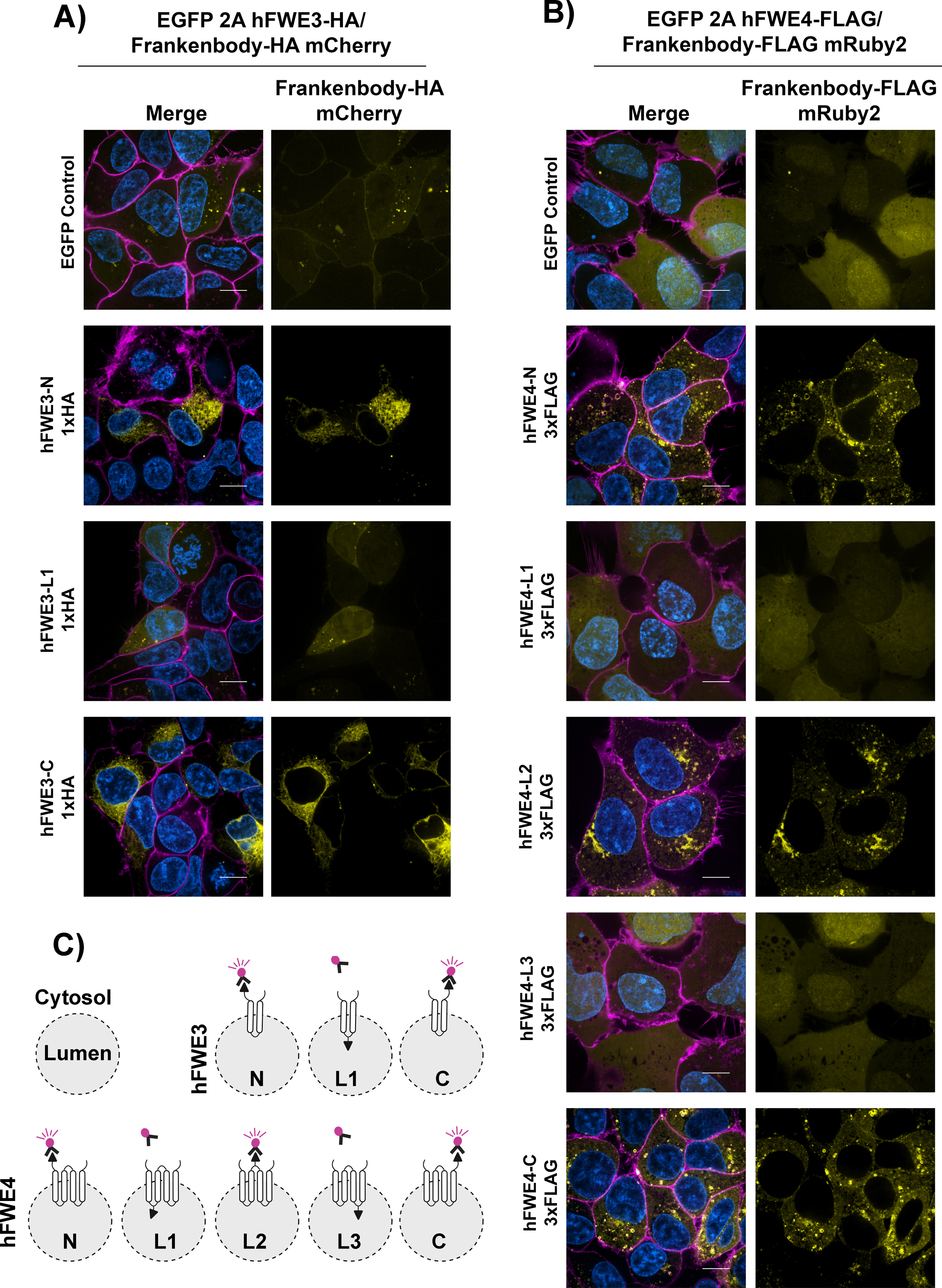

Supplement: Supporting Figure S10 — Frankenbodies demonstrate unique subcellular localizations of hFWE3 and hFWE4. HEK293 cells stably expressing EGFP alone, or coexpressing non-fused EGFP and hFWE3 (A) or hFWE4 (B) harboring epitope tags at indicated positions were transduced with lentivirus encoding HA- or FLAG-targeting Frankenbodies. Cells were live imaged for native mCherry/mRuby2 fluorescence (yellow), CellMask Plasma Membrane label (magenta), and Hoechst (blue). Representative single Z-slices are presented from 100× live cell confocal images, scale bar = 10 μm. C, schematic overview of hFWE isoform structure on intracellular membranes, with hFWE4 assuming a four transmembrane pass structure and hFWE3 assuming a two transmembrane pass structure. Both isoforms position N and C termini cytosolically. [file figs10.jpg]
